# The vaginal microbiome and preterm birth

**DOI:** 10.1038/s41591-019-0450-2

**Published:** 2019-05-29

**Authors:** Jennifer M. Fettweis, Myrna G. Serrano, J. Paul Brooks, David J. Edwards, Philippe H. Girerd, Hardik I. Parikh, Bernice Huang, Tom J. Arodz, Laahirie Edupuganti, Abigail L. Glascock, Jie Xu, Nicole R. Jimenez, Stephany C. Vivadelli, Stephen S. Fong, Nihar U. Sheth, Sophonie Jean, Vladimir Lee, Yahya A. Bokhari, Ana M. Lara, Shreni D. Mistry, Robert A. Duckworth, Steven P. Bradley, Vishal N. Koparde, X. Valentine Orenda, Sarah H. Milton, Sarah K. Rozycki, Andrey V. Matveyev, Michelle L. Wright, Snehalata V. Huzurbazar, Eugenie M. Jackson, Ekaterina Smirnova, Jonas Korlach, Yu-Chih Tsai, Molly R. Dickinson, Jamie L. Brooks, Jennifer I. Drake, Donald O. Chaffin, Amber L. Sexton, Michael G. Gravett, Craig E. Rubens, N. Romesh Wijesooriya, Karen D. Hendricks-Muñoz, Kimberly K. Jefferson, Jerome F. Strauss, Gregory A. Buck

**Affiliations:** 1 Department of Microbiology and Immunology, School of Medicine, Virginia Commonwealth University, Richmond, VA USA; 2 Department of Obstetrics and Gynecology, School of Medicine, Virginia Commonwealth University, Richmond, VA USA; 3 Center for Microbiome Engineering and Data Analysis, Virginia Commonwealth University, Richmond, VA USA; 4 Supply Chain Management and Analytics, School of Business, Virginia Commonwealth University, Richmond, VA USA; 5 Department of Statistical Sciences and Operations Research, College of Humanities and Sciences, Virginia Commonwealth University, Richmond, VA USA; 6 Department of Computer Science, College of Engineering, Virginia Commonwealth University, Richmond, VA USA; 7 VCU Life Sciences, Virginia Commonwealth University, Richmond, VA USA; 8 Division of Neonatal Medicine, School of Medicine, Virginia Commonwealth University, Richmond, VA USA; 9 Department of Pediatrics, School of Medicine, Children’s Hospital of Richmond at Virginia Commonwealth University, Richmond, VA USA; 10 Department of Chemical and Life Science Engineering, College of Engineering, Virginia Commonwealth University, Richmond, VA USA; 11 Center for the Study of Biological Complexity, VCU Life Sciences, Virginia Commonwealth University, Richmond, VA USA; 12 School of Medicine, Virginia Commonwealth University, Richmond, VA USA; 13 Nell Hodgson Woodruff School of Nursing, Emory University, Atlanta, GA USA; 14 Department of Women’s Health, Dell School of Medicine, University of Texas at Austin, Austin, TX USA; 15 School of Nursing, University of Texas at Austin, Austin, TX USA; 16 Department of Biostatistics, School of Public Health, West Virginia University, Morgantown, WV USA; 17 Department of Mathematical Sciences, University of Montana, Missoula, MT USA; 18 Department of Biostatistics, School of Medicine, Virginia Commonwealth University, Richmond, VA USA; 19 Pacific Biosciences, Menlo Park, CA USA; 20 Global Alliance to Prevent Prematurity and Stillbirth, Seattle, WA USA; 21 Department of Obstetrics & Gynecology, University of Washington, Seattle, WA USA

**Keywords:** Microbiome, Urogenital reproductive disorders, Predictive markers

## Abstract

The incidence of preterm birth exceeds 10% worldwide. There are significant disparities in the frequency of preterm birth among populations within countries, and women of African ancestry disproportionately bear the burden of risk in the United States. In the present study, we report a community resource that includes ‘omics’ data from approximately 12,000 samples as part of the integrative Human Microbiome Project. Longitudinal analyses of 16S ribosomal RNA, metagenomic, metatranscriptomic and cytokine profiles from 45 preterm and 90 term birth controls identified harbingers of preterm birth in this cohort of women predominantly of African ancestry. Women who delivered preterm exhibited significantly lower vaginal levels of *Lactobacillus crispatus* and higher levels of BVAB1, *Sneathia amnii*, TM7-H1, a group of *Prevotella* species and nine additional taxa. The first representative genomes of BVAB1 and TM7-H1 are described. Preterm-birth-associated taxa were correlated with proinflammatory cytokines in vaginal fluid. These findings highlight new opportunities for assessment of the risk of preterm birth.

## Abstract (web summary)

As part of the second phase of Human Microbiome Project, the Multi-Omic Microbiome Study: Pregnancy Initiative presents a community resource to help better understand how microbiome and host profiles change throughout pregnancy as well as to identify new opportunities for assessment of the risk of preterm birth.

## Main

Approximately 15 million preterm births at less than 37 weeks of gestation occur annually worldwide^1^. Preterm birth (PTB) remains the second most common cause of neonatal death across the globe, and the most common cause of infant mortality in middle- and high-income economies^2^. The consequences of PTB persist from early childhood into adolescence and adulthood^3,4^. In the United States, striking population differences with respect to PTB exist, with women of African ancestry having a substantially larger burden of risk. The estimated annual cost of PTB in the United States alone is over US$26.2 billion^5^. Despite these statistics, there remains a paucity of effective strategies for predicting and preventing PTB.

Although maternal and fetal genetics, and gene–environment interactions, clearly play roles in determining the length of gestation, environmental factors, including the microbiome, are the most important contributors to PTB, particularly among women of African ancestry^6^. Microbe-induced inflammation resulting from urinary tract infection, sexually transmitted infections, including trichomoniasis, or bacterial vaginosis is thought to be a cause of PTB^7,8^. Ascension of microbes^7,9^ from the lower reproductive tract to the placenta, fetal membranes and uterine cavity, and hematogenous spread of periodontal pathogens from the mouth, have also been invoked to explain the up to 40–50% of preterm births that are associated with microbial etiologies^10,11^.

A homogeneous *Lactobacillus*-dominated microbiome has long been considered the hallmark of health in the female reproductive tract. In contrast, a vaginal microbiome with high species diversity, as observed with bacterial vaginosis, has been associated with increased risk for acquisition and transmission of sexually transmitted infections, PTB and pelvic inflammatory disease^12–15^. However, many asymptomatic healthy women have diverse vaginal microbiota. More refined approaches are needed to assess risk, promote health, and prevent and treat disease^16–21^.

Recent reports of the microbiome in pregnant women^22–39^ have suggested that the composition of the vaginal microbiome has a significant population-specific impact on PTB risk. Several studies that focused on populations predominantly of European descent^22–25^ have associated *Lactobacillus crispatus* with a lower risk of PTB, and the finding was replicated in a cohort of predominantly African descent^25^. As first reported by Ravel et al.^16^, and subsequently confirmed in other studies^21,40^, the vaginal microbiome profiles of women of African and European ancestry differ significantly. Although distinct taxa have been associated with PTB in women of African ancestry in some studies^25,26^, others have not found significant associations^27,30^. Women of African descent are less likely to exhibit vaginal lactobacilli, frequently have vaginal *L. crispatus* predominance and are more likely to exhibit increased vaginal microbial diversity^16,21^. Consequently, population-specific studies may be required to assess the broad impacts of the vaginal microbiome on risk of PTB and to identify contributing taxa that may be carried by only a small subset of women.

In the present study, we report a community resource that includes samples collected longitudinally during 1,572 pregnancies of women from diverse ancestries, and omics data generated from samples collected from 597 pregnancies in a collaborative effort under the umbrella of the National Institutes of Health’s integrative Human Microbiome Project (iHMP)^41^. Furthermore, we provided an analysis of the longitudinal, comprehensive, multi-omic profiling of vaginal samples from 45 women who experienced spontaneous PTB and 90 case-matched controls, in a cohort of women of predominantly African ancestry. In an initial analysis of this dataset, which represents one of the largest and most comprehensive studies of the vaginal microbiome to date, we identified vaginal microbial signatures in women who went on to experience PTB.

## Results

### The Multi-Omic Microbiome Study: Pregnancy Initiative

**Fig. 1:**
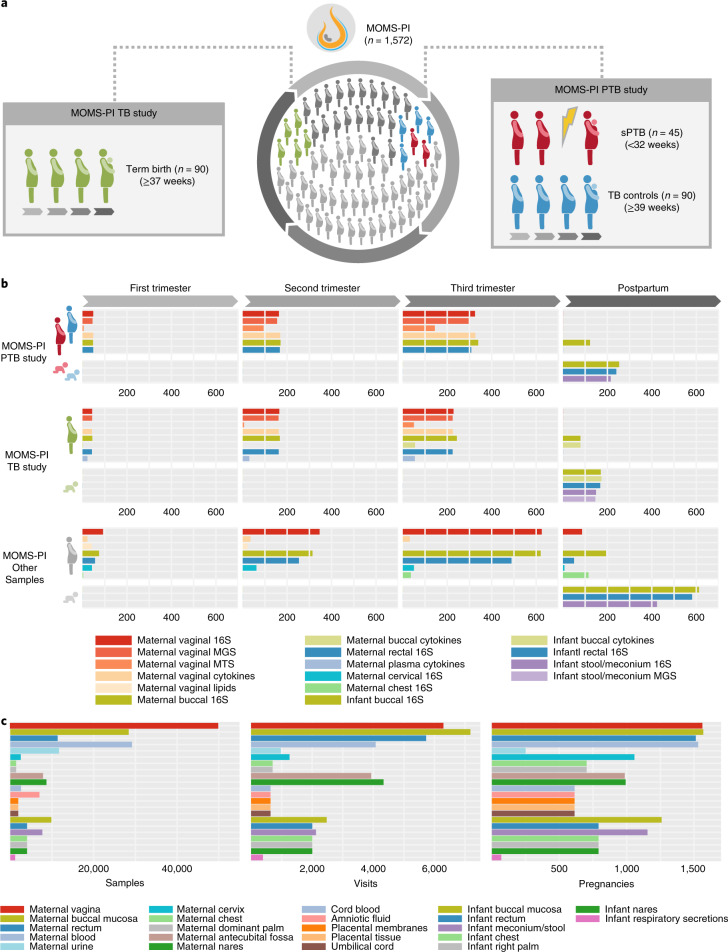
MOMS-PI resources. **a**, An overview of the study designs for the MOMS-PI PTB study (45 spontaneous preterm (sPTB) cases and 90 term controls) and the MOMS-PI TB study (90 women who delivered at term or early term and their neonates). Both cohorts were selected from the phase 1 RAMS Registry cohort (*n* = 627). **b**, Omics data were generated from samples from the MOMS-PI PTB and MOMS-PI TB studies and 384 additional pregnancies from the overall MOMS-PI cohort. Samples from the 12 women who were selected for both the MOMS-PI PTB study and the MOMS-PI TB study are depicted under both studies. Omics data types include 16S rRNA amplicon sequencing, metagenomic sequencing (MGS), metatranscriptomic sequencing (MTS), host cytokine assays and lipidomics. **c**, A total of 206,437 samples were collected at more than 7,000 visits from 1,572 pregnancies in the MOMS-PI study, and are archived in the RAMS Registry.

The longitudinal iHMP study, the Multi-Omic Microbiome Study: Pregnancy Initiative (MOMS-PI) includes a total of 1,572 pregnancies, with 992 pregnancies from clinics associated with the Research Alliance for Microbiome Science (RAMS) Registry, based at Virginia Commonwealth University (VCU) in Virginia, and 580 pregnancies from sites associated with the Global Alliance to Prevent Prematurity and Stillbirth (GAPPS) in Washington State. The resource features two comprehensive datasets of integrated microbiome and host functional properties measured longitudinally in pregnancy and the perinatal period (Fig. 1): (1) the MOMS-PI Preterm Birth (PTB) study dataset generated from a case–control study of 45 women predominantly of African ancestry, who delivered spontaneously preterm, and 90 case-matched women who delivered at term; and (2) the MOMS-PI Term Birth (TB) study dataset generated from an ethnically diverse retrospective cohort study of 90 women, who delivered at term or early term^42^. From a selection of 12,039 samples from 597 pregnancies, we generated: (1) 16S ribosomal RNA (rRNA) taxonomic profiles from 6,452 samples from pregnant women and 2,753 samples from neonates; (2) metagenome profiles from 930 samples from pregnant women and 146 samples from neonates; (3) metatranscriptome profiles from 297 samples from pregnant women; (4) cytokine profiles from 1,223 samples from pregnant women and 173 samples from neonates; and (5) lipid profiles from 63 samples from pregnant women. In the overall MOMS-PI study, we collected a total of 206,437 samples from pregnant women and their neonates, which have been archived in the RAMS Registry (see ramsregistry.vcu.edu) (Fig. 1c). Comprehensive health history and outcome data were also collected longitudinally.

### Vaginal microbiome profiles show PTB-associated trends

In the present study, we focus our analysis on a comprehensive multi-omic profiling of vaginal samples in the MOMS-PI PTB study. We analyzed 45 single gestation pregnancies that met the criteria for spontaneous PTB (23–36 weeks 6 days of gestational age) and 90 single gestation pregnancies that extended through term (≥39 weeks) to avoid issues possibly associated with early term births^43–45^. The TB controls in the MOMS-PI PTB study were case matched to the PTB group (2TB:1PTB) for age, race and annual household income. On average, the earliest samples were collected at 18 weeks of gestation, and the mean number of sampling visits per participant was 7. The respective mean and median gestational age at delivery was 34, 0/7 and 35, 6/7 for the PTB group and 40, 0/7 and 39, 6/7 for the TB group.

**Fig. 2:**
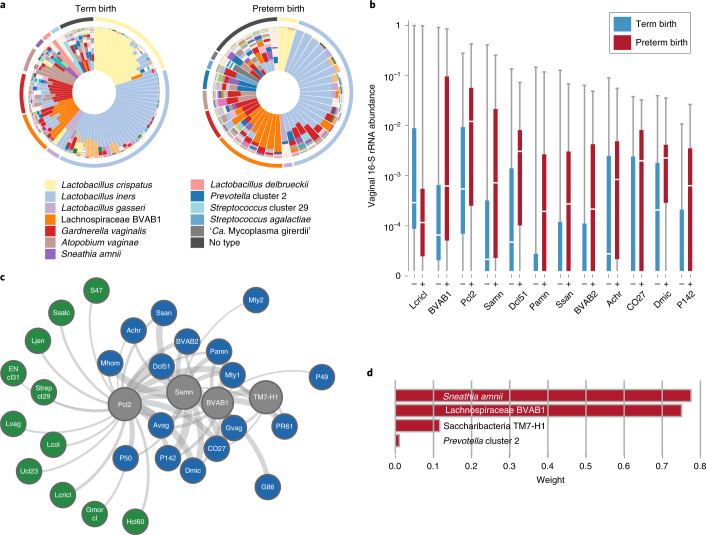
Bacterial taxa associated with spontaneous PTB. **a**, Vagitypes of 90 women who delivered at term (≥39 weeks of gestation), and 45 women who delivered prematurely (<37 weeks of gestation) showing 13 community states, or vagitypes. **b**, Abundance of taxa significantly different in PTB (*n* = 45) and TB (*n* = 90) cohorts. These taxa have *P* <0.05 for the Mann–Whitney *U*-test (two-sided) for difference in proportional abundance between the cohorts, corrected using the Benjamini–Hochberg procedure with an FDR of 5%. TB is indicated in blue as (–) and PTB in red as (+). Boxes show the median and interquartile range; whiskers extend from minimum to maximum values within each cohort. **c**, Network analysis of four taxa highly associated with PTBs. Negative correlations are shown in green, positive correlations in blue and predictive taxa in gray. Edge weights represent the strength of correlation. See Supplementary Table 3 for abbreviations. **d**, Predictive linear model for PTBs that produces a score based on weighted log(abundances) of four taxa in vaginal 16S rRNA profiles in the 6- to 24-week gestational age range. Taxa abbreviations: Lcricl, *L. crispatus* cluster; BVAB1, Lachnospiraceae BVAB1; Pcl2, *Prevotella* cluster 2; Samn, *S. amnii*; Dcl51, *Dialister* cluster 51; Pamn, *P. amnii*; BVAB2, Clostridiales BVAB2; CO27, Coriobacteriaceae OTU27; Dmic, *Dialister micraerophilus*; P142, *Parvimonas* OTU142.

**Extended Data Figure 1:**
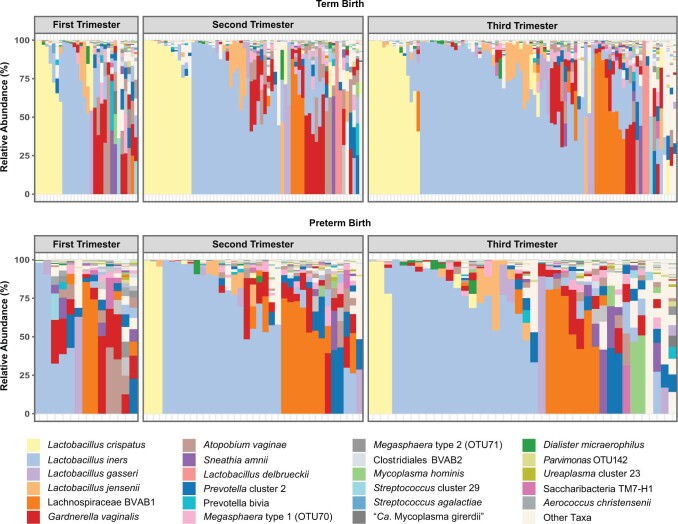
Species-level vaginal microbiome composition in women who experience TB or PTB. Stacked bar charts illustrating the vaginal microbiome profiles from 16S rRNA surveys of one sample per trimester from each pregnancy. Samples are ordered according to decreasing relative abundance. Twenty-nine abundant taxa of interest are shown with all others pooled into ‘Other’.

The cohort predominantly comprised women of African ancestry (~78%), with a median annual income of less than US$20,000 and an average age of 26 years (Table 1, and see Supplementary Table 1). Microbiome profiles of the first vaginal samples collected at study enrollment (Fig. 2a, and see Extended Data Fig. 1) were generated by 16S rRNA taxonomic analysis. For vaginal samples, the dominant bacterial taxon is one clinically meaningful measure by which to stratify samples^16,46^. Women who went on to deliver at term were more likely to exhibit *L. crispatus* predominance in the vaginal microbiome (*P* = 0.014, Fig. 2a,b, and see Supplementary Table 2a and Extended Data Fig. 1), paralleling earlier observations^17,22–25^. A Markov chain analysis to assess vagitype changes throughout pregnancy did not reveal statistically significant differences in transition rates between case and control groups. However, point estimates of probabilities of transition to the BV-associated bacterium 1 (BVAB1) vagitype were higher in the PTB group, whereas point estimates of transition to the *L. crispatus* group were higher in the term group, although the differences failed to reach significance (see Supplementary Table 2b).

**Table 1:** Description of cohort studied in this project

	Preterm delivery <37 weeks (*n* = 45)	Term delivery ≥39 weeks (*n* = 90)
Mean age (years)^a^	26 (5.68)	25.9 (5.43)
Ancestry/ethnicity (no. (%))		
African	35 (77.8)	71 (78.9)
European	6 (13.3)	13 (14.4)
Hispanic	3 (6.7)	5 (5.6)
Native American	1 (2.2)	1 (1.1)
Household income (no. (%))^b^		
<US$20,000	29 (72.5)	66 (77.7)
US$20,000–59,999	9 (22.5)	15 (17.6)
US$60,000+	2 (5.0)	4 (4.7)
Vaginal delivery (no. (%))	38 (84.4)	74 (82.2)
Previous preterm (no. (%))	14 (31.1)	9 (10.0)
Preterm premature rupture of the membranes (no. (%))	26 (57.8)	0 (0)

^a^Standard deviation listed in parentheses.

^b^Missing values *n* = 5 (PTB), *n* = 5 (TB).

**Extended Data Figure 2:**
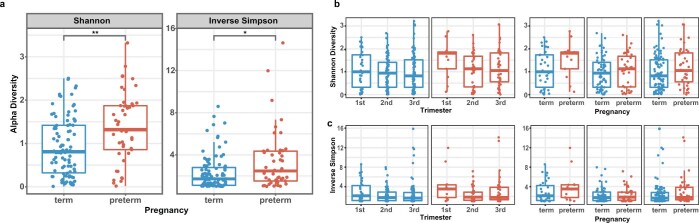
Alpha diversity measures for PTB and TB cohorts. **a**, Shannon index and inverse Simpson index for the cross-sectional cohort in Fig. 2 (PTB *n* = 45, TB *n* = 90) shows that alpha diversity is significantly higher (*P* = 0.0026, *P* = 0.12, respectively) in the preterm cohort using two-sided Wilcoxon’s test followed by a *P* value adjustment using Bonferroni’s correction with 5% FDR. Boxes show median and interquartile range; whiskers extend from minimum to maximum values within each cohort. **b**,**c** Shannon index (**b**) and inverse Simpson index (**c**) diversity measures are shown with comparisons by trimester (TB first trimester: *n* = 30; PTB first trimester: *n* = 13; TB second trimester: *n* = 64; PTB second trimester: *n* = 35; TB third trimester: *n* = 90; PTB third trimester: *n* = 40). The Kruskal–Wallis two-sided test (analysis of variance) was used followed by post-hoc pairwise comparison with Bonferroni’s correction and 5% FDR. Comparison within trimesters was performed using two-sided Wilcoxon’s test with Bonferroni’s *P* value adjustment. Boxes show median and interquartile range; whiskers extend from minimum to maximum values within each cohort. For the trimester comparisons, no tests were significant.

**Extended Data Figure 3:**
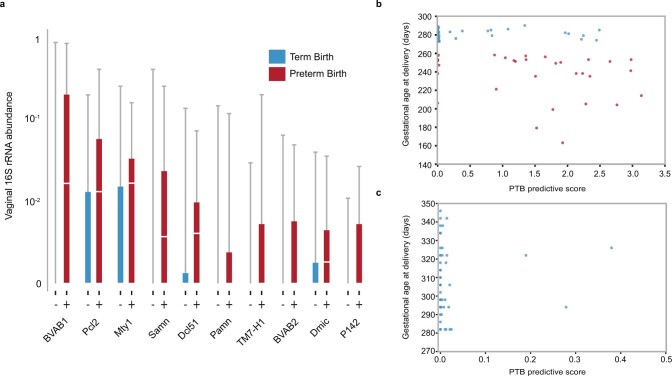
Taxa that significantly differ in PTB and TB cohorts. The distributions of proportional abundance of taxa greatly differ in PTB (*n* = 31) and TB (*n* = 59) cohorts; the earliest sample available for each subject within the first 24 weeks of pregnancy was used for each subject. Abundance values below 0.001 were rounded down to 0. The taxa are: BVAB1: Lachnospiraceae BVAB1, Pcl2: *Prevotella* cluster 2, Mty1: *Megasphaera* OTU70 type1, Samn: *Sneathia amnii*, TM7: TM7-H1, Dcl51: *Dialister* cluster 51, Pamn: *Prevotella amnii*, BVAB2: Clostridiales BVAB2, Dmic: *Dialister micraerophilus* and P142: *Parvimonas* OTU142. These 10 taxa have *P* <0.05 to support a significant difference in proportional abundance between PTB and TB cohorts using a Mann–Whitney *U*-test (two-sided) and the Benjamini–Hochberg correction procedure with an FDR of 5%. Boxes show median and interquartile range; whiskers extend from minimum to maximum values within each cohort. **b**,**c**, Scatter plot of the PTB predictive score returned by the model (horizontal axis) plotted against gestational age at birth (vertical axis). Each point corresponds to a sample from a subject: red, PTB subjects (*n* = 31); blue, TB subjects (*n* = 59). **c**, Shows more detailed view of the region where most (48 of 59) of the TB samples are located.

Overall diversity was increased in samples from women who would go on to experience PTB (see Extended Data Fig. 2), and 12 taxa showed a significant difference in abundance between the PTB and the TB groups (Fig. 2b). *L. crispatus* was greatly reduced in PTB samples, and several other taxa, including BVAB1, *Prevotella* cluster 2 and *Sneathia amnii*, were more abundant in PTB samples (*q* < 0.05; Fig. 2b, and see Supplementary Tables 4 and 5). *Prevotella* cluster 2 comprises several closely related taxa of that genus^47^, including *Prevotella timonensis* and *Prevotella buccalis*. Through an analysis of samples collected from the 31 PTB and 59 TB subjects who had samples collected early (6–24 weeks of gestational age) in pregnancy, we identified two additional taxa that were significantly increased in PTB samples: *Megasphaera* type 1 and TM7-H1 (that is, BVAB-TM7) (see Extended Data Fig. 3). Both of these taxa have been previously associated with adverse conditions of vaginal health^12^. These findings extend those of a previous study that found carriage of BVAB1 and *Sneathia* species in early and mid-pregnancy to be associated with spontaneous PTB^26^. To our knowledge, this is the first report of an association of TM7-H1 with PTB.

Early prediction of risk for PTB is critical for the development of new strategies for prevention and intervention. As a proof of concept, we developed a model for identifying the most discriminative taxa for PTB using 16S rRNA data from samples collected at 24 weeks of gestation or earlier. Model construction involved selecting taxa that are differentially represented in the cohorts as assessed using the Mann–Whitney *U*-test (Fig. 2d, and see Extended Data Fig. 3a and Supplementary Table 5), and assigning weights to these taxa using L_1_-regularized logistic regression. The resulting model incorporates four taxa: *S. amnii*, BVAB1, *Prevotella* cluster 2 and TM7-H1, which are all positively correlated with PTB (Fig. 2d, and see Extended Data Fig. 3b,c). The discriminative model is significant (*P* = 0.0024) and has an expected sensitivity of 77.4%, specificity of 76.3%, and an area under the receiver operating characteristics (AUROC) curve of 0.723 for samples not used during training. This model, based on microbiome composition data, had 5–7% greater sensitivity and specificity than a model constructed using only clinical variables with a slight reduction in the AUROC curve (that is, 0.723 versus 0.764). A network analysis of these four taxa (Fig. 2c) shows them to be positively correlated with taxa associated with vaginal dysbiosis.

**Fig. 3:**
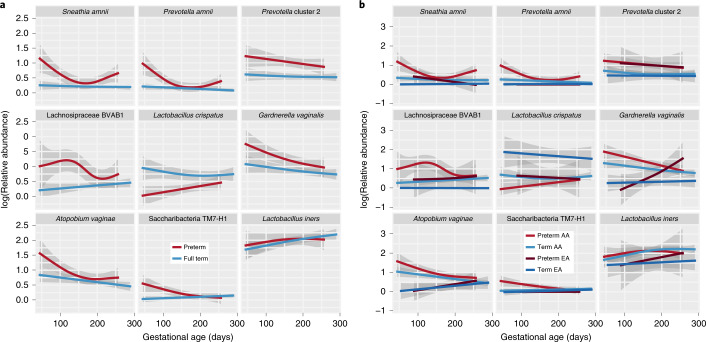
Longitudinal GAMM of vaginal microbiome composition during pregnancy. The model incorporates BMI, vaginal pH, pregnancy outcome (PTB, TB), a smoother for gestational age and a random subject effect to longitudinally model log-transformed relative abundances of vaginally relevant taxa (see  Methods). Each figure plots log-transformed abundances of taxa throughout pregnancy. **a**, Plots comparing the PTB case (*n* = 41) and TB cohorts (*n* = 90). **b**, Comparison of the results from preterm and full-term women of African and European ancestry (that is, EA PTB, *n* = 7; AA PTB, *n* = 31; EA TB, *n* = 13; AA TB, *n* = 73). Confidence intervals (98%) are shown.

We further examined longitudinal trends of key taxa using a generalized additive mixed effect model (GAMM) incorporating body mass index (BMI), vaginal pH, ethnicity and preterm status (Fig. 3a, and see Supplementary Fig. 1a). Many taxa identified as associated with PTB in the 16S rRNA cross-sectional analyses, including *S. amnii* (*P* = 0.0015), *Prevotella* cluster 2 (*P* = 0.0031), BVAB1 (*P* = 0.0037) and *P. amnii* (*P* = 0.0031), were also associated with PTB in this longitudinal analysis. Women who delivered preterm experienced large decreases during pregnancy in *S. amnii* (*P* = 0.0163), BVAB1 (*P*= 0.0002), *P. amnii* (*P* = 0.0004), *Gardnerella vaginalis* (*P* = 0.0074), TM7-H1 (*P* = 0.0005) and *Atopobium vaginae* (*P* = 0.0090). *Prevotella* cluster 2 also showed a decreased prevalence later in pregnancy, but the decrease was not statistically significant. In contrast, *L. crispatus* increased in prevalence (*P* = 0.0320) over the course of the pregnancy in women who delivered preterm. Women who delivered at term exhibited significant decreases in prevalence of *A. vaginae* (*P* < 0.0001) and *G. vaginalis* (*P* = 0.0012), and an increase in *L. iners* (*P* = 0.0273).

A stratification of the longitudinal GAMM analysis by ancestry (Fig. 3b, and see Supplementary Fig. 1b) showed that, over the duration of pregnancy, women of African ancestry who delivered preterm experienced significant decreases in the prevalence of *A. vaginae* (*P* = 0.0011), BVAB1 (*P* = 0.0003), *G. vaginalis* (*P* = 0.0002), *P. amnii* (*P*= 0.0013), *S. amnii* (*P* = 0.0219) and TM7-H1 (*P* = 0.0014). Women of African ancestry (AA) who delivered at term exhibited fewer changes in the modeled taxa throughout pregnancy, although decreases in *A. vaginae* (*P* = 0.0001) and *G. vaginalis* (*P* = 0.0003) and an increase in *L. iners* (*P* = 0.0404) were observed. Women of European ancestry (EA) generally exhibited stable microbiome profiles during pregnancy, although an increase in prevalence of *G. vaginalis* (*P* = 0.0401) was noted for women who delivered preterm. *G. vaginalis* has been previously reported as a microbial signature for PTB in cohorts of women of predominantly European ancestry^25^. Overall, our observations are consistent with previous reports of dynamic changes in the vaginal microbiome in pregnancy^18,48,49^ and results from the MOMS-PI TB study, which show that the dynamics of vaginal microbiome differ by ancestry, with women of African ancestry exhibiting a more pronounced decrease in microbial diversity throughout the course of a term pregnancy^42^.

**Extended Data Figure 4:**
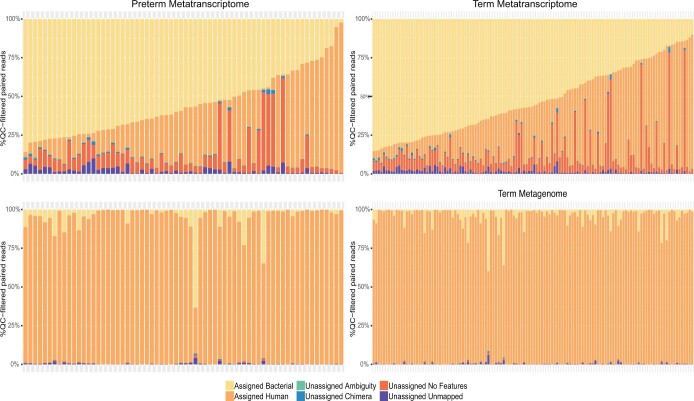
Assignment of metatranscriptomics and metagenomics quality-filtered reads in PTB and TB cohorts. Non-human reads were mapped to a custom database corresponding to 56 bacterial taxa. Values are shown as the percentage abundance.

**Extended Data Figure 5:**
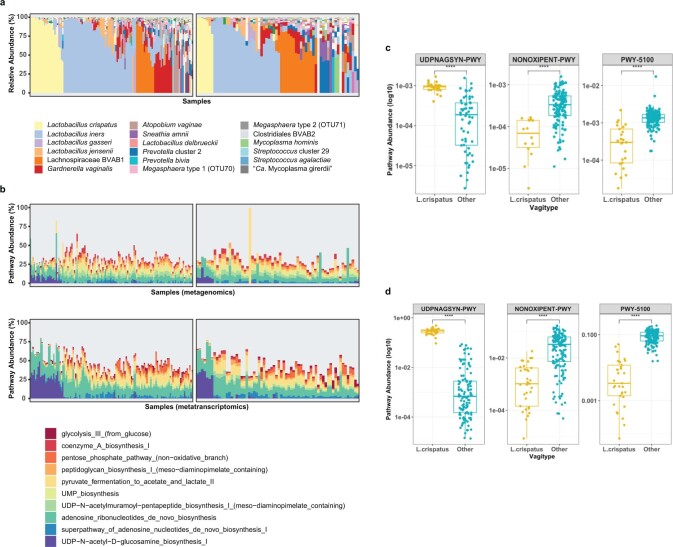
Metabolic pathway abundance by vagitype. **a**, Stacked barplots showing the vaginal microbial profiles from 16S rRNA surveys; **b**, the metabolic pathway abundances from paired MGS (top panel) and MTS (bottom panel) of vaginal samples collected during pregnancies of 90 women who delivered at term (≥39 weeks) (left) and 45 women who delivered preterm (<37 weeks) (right). Results show the top 10 metabolic pathways calculated by HUMAnN2. **c**,**d**, The presence of the UDP-*N*-acetyl-d-glucosamine biosynthesis pathway and non-oxidative branch of pentose phosphate pathway differ between *L. crispatus* and other vagitypes as measured by MGS data (**c**) (*L. crispatus* UDPNAGSYN: *n* = 32, Other UDPNAGSYN: *n* = 59; *L. crispatus* NONOXYPENT-PWY: *n* = 14, Other NONOXYPENT-PWY: *n* = 138; *L. crispatus* PWY-5100: *n* = 25,Other PWY-5100: *n* = 156) and MTS data (**d**) (*L. crispatus* UDPNAGSYN: *n* = 33, Other UDPNAGSYN: *n* = 117; *L. crispatus* NONOXYPENT-PWY: *n* = 33, Other NONOXYPENT-PWY: *n* = 157; *L. crispatus* PWY-5100: *n* = 33,Other PWY-5100: *n* = 159). Two-sided Wilcoxon’s *P* test (*P* <0.05) with Bonferroni’s adjustment at a 5% FDR threshold was used. Boxes show median and interquartile range; whiskers extend from minimum to maximum values within each cohort.

A total of 496 longitudinal vaginal samples from participants in the MOMS-PI PTB study (see Fig. 1b) were subjected to metagenomic sequencing (MGS) and a subset of 243 samples was subjected to metatranscriptomic sequencing (MTS) (see Extended Data Fig. 4). At the pathway level, the functional and metabolic potentials of the microbial communities were largely conserved, with the exception of *L. crispatus*-dominated samples, which exhibited a much higher proportional metabolic potential and transcriptional activity of the UDP-*N*-acetyl-d-glucosamine (UDP-GlcNAc) biosynthesis pathway (see Extended Data Fig. 5). UDP-GlcNAc is a precursor for peptidoglycan, one of the best-described, microbe-associated molecular patterns involved in the modulation of host cytokine production via toll-like receptor signaling^50^. Additional studies are required to determine whether combinations of microbe-associated molecular patterns produced by the vaginal microbiota modulate host cytokine levels and impact urogenital health. In contrast, the proportional transcriptional activities of genes classified to the pathway of pyruvate fermentation to acetate and lactate II, and the non-oxidative branch of the pentose phosphate pathway, were lower in *L. crispatus*-dominated samples (see Extended Data Fig. 5). This finding is consistent with reports of reduced levels of lactic acid and increased concentrations of short-chain fatty acids in vaginal samples of women with bacterial vaginosis^51^. Short-chain fatty acids have been suggested to reduce antimicrobial activity and promote proinflammatory cytokines in the vaginal environment.

### Metagenomic assembly of reference genomes of bacterial taxa associated with PTB

MGS data generated with Pacific BioSciences and Illumina sequencing technologies were used to generate the first genomes of TM7-H1 (CP026537) and BVAB1 (PQVO000000), respectively. BVAB1, with a genome of ~1.45 megabases (Mb), is classified to the Family Lachnospiraceae of the Order Clostridiales, and is not closely related to any other known bacterium (see Supplementary Table 6). TM7-H1, with a genome of ~0.72 Mb, falls into the Phylum *Candidatus* Saccharibacteria and exhibits only ~66% nucleotide identity with the recently described oral TM7x isolate (NZ_CP007496)^52^. TM7-H1 encodes a putative α-amylase and is predicted to be able to utilize glycogen as a carbon source (see Supplementary Data 1). Similar to TM7x^52^, TM7-H1 lacks de novo biosynthetic capabilities for essential amino acids (see Supplementary Table 7), and likely depends on other organisms in the vaginal environment for survival. However, although TM7x is an obligate parasitic epibiont, it remains unknown whether TM7-H1 similarly lives on the surface of another bacterial species in the vaginal environment. We identified 243 and 421 metabolic reactions, respectively, in TM7-H1 and BVAB1 (see Supplementary Tables 7 and 8, and Supplementary Data 2). Both organisms are predicted to have the ability to produce pyruvate, acetate, l-lactate and propionate. BVAB1 encodes additional pathways for production of acetaldehyde, d-lactate, formate and acetyl-CoA. Neither is predicted to have a functional tricarboxylate cycle, and TM7-H1 completely lacks genes related to butyrate metabolism. As described above, production of short-chain fatty acids has been linked to a proinflammatory state^51^, with possible implications for disease.

### Bacterial taxa associated with PTB in metagenomic and metatranscriptomic data

**Extended Data Figure 6:**
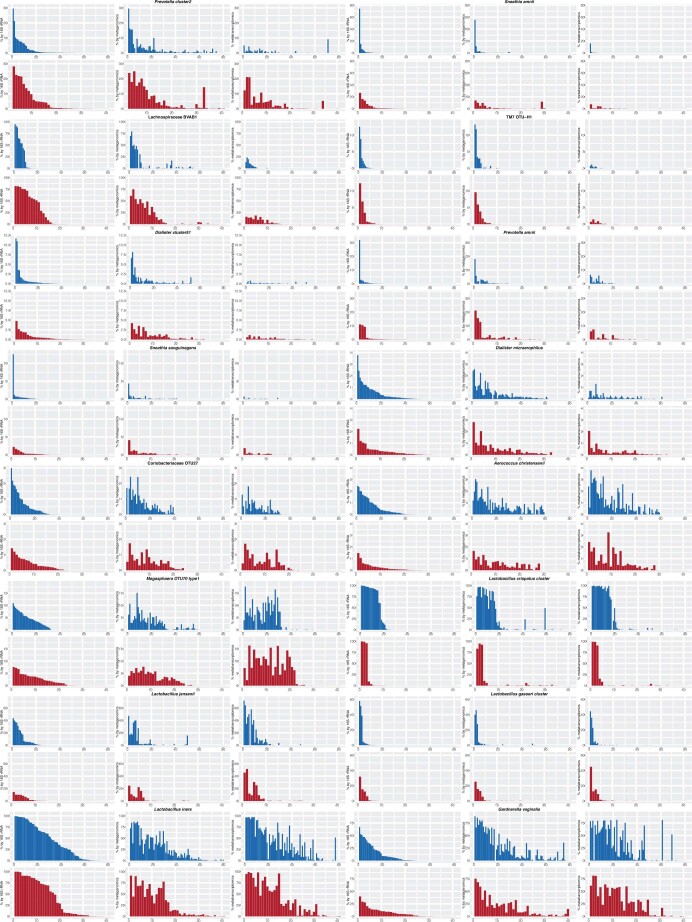
Comparison of the proportional abundance of taxa by 16S rRNA (left), metagenomics (middle) and metatranscriptomics (right) in term (blue) and preterm (red) cohorts. Proportional abundance is shown relative to the 56 bacterial taxa that were measured across all three assays. The 16S rRNA, metagenomics and metatranscriptomics measures from PTB (*n* = 41) and TB (*n* = 81) were from a single time point per participant. The *y* axis is scaled based on the maximum proportional abundance for the taxon.

On average, approximately 95% of MGS reads and 30% of MTS reads were identified as human (see Extended Data Fig. 4). Most non-human MGS and MTS reads mapped to our customized vaginal bacterial database, with only a small fraction remaining unmapped (that is, average of 0.45% full-term metagenomics, 0.41% preterm metagenomics, 1.67% full-term metatranscriptomics and 2.46% preterm metatranscriptomics) (see Supplementary Data 3). We compared the relative proportional abundance of taxa from the 16S rRNA assay with the relative proportional abundance of metagenomic and metatranscriptomic data that mapped to non-ribosomal genes across the 56 taxa in our database. Although proportional differences were observed across detection methods, there was concordance in detection of taxa using 16S rRNA profiles, MGS and MTS (see Extended Data Fig. 6).

**Fig. 4:**
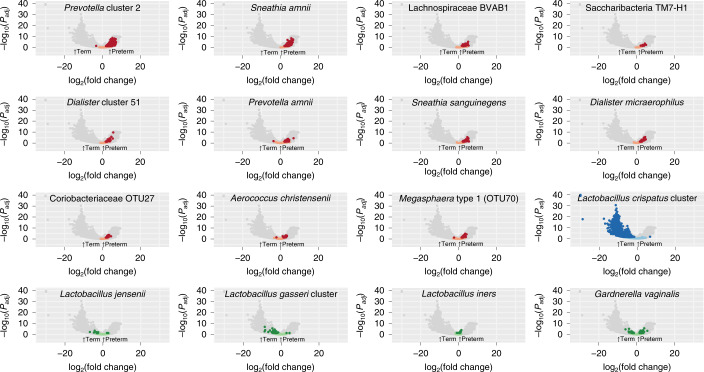
Volcano plot depicting transcripts that differ greatly between term and preterm cohorts in metatranscriptomics data for taxa of interest. Analysis was performed by mapping reads from PTB (*n* = 41) and TB (*n* = 81) samples to a custom database of genomes representing 56 taxa. Comparative analysis was performed with DESeq2 using a global scaling approach. Genes in candidate PTB taxa identified in 16S rRNA analyses that differ significantly at *P*_adj _< 0.05 with a two-sided Wald test, as implemented in DESeq2 with a Benjamini–Hochberg FDR correction, are shown in red and those that were not statistically significant are shown in pink. Genes are also shown for the *L. crispatus* cluster (associated with TB in the 16S rRNA analyses), with genes that differ significantly (*P*_adj_ < 0.05) in transcript levels shown in dark blue and those that do not in light blue. Four other common and abundant taxa (*L. jensenii, L. gasseri* cluster, *L. iners* and *G. vaginalis*) are shown, with dark green dots denoting genes that differ significantly (*P*_adj_ < 0.05) between cohorts and light green dots denoting those that were not statistically significant.

Paired MGS and MTS data were available for 41 women who delivered preterm and for 81 term controls. Thus, we analyzed a single time point per participant, with a mean gestational age of sampling at 25 weeks for the preterm cohort and 26 weeks for the full-term cohort; we also used a global scaling approach to normalize to all genes in the 56 taxa in our database. In the preterm samples, we observed higher transcript levels of genes from all of the taxa identified as candidate markers of PTB that were analyzed, with inclusion of samples collected even later in pregnancy than those used for 16S rRNA analyses (Fig. 4). Conversely, we observed higher transcript levels of *L. crispatus* in the term samples. *L. jensenii*, *L. gasseri*, *L. iners* and *G. vaginalis* had relatively few genes that showed very different transcript levels between the term and preterm cohorts (Fig. 4).

**Extended Data Figure 7:**
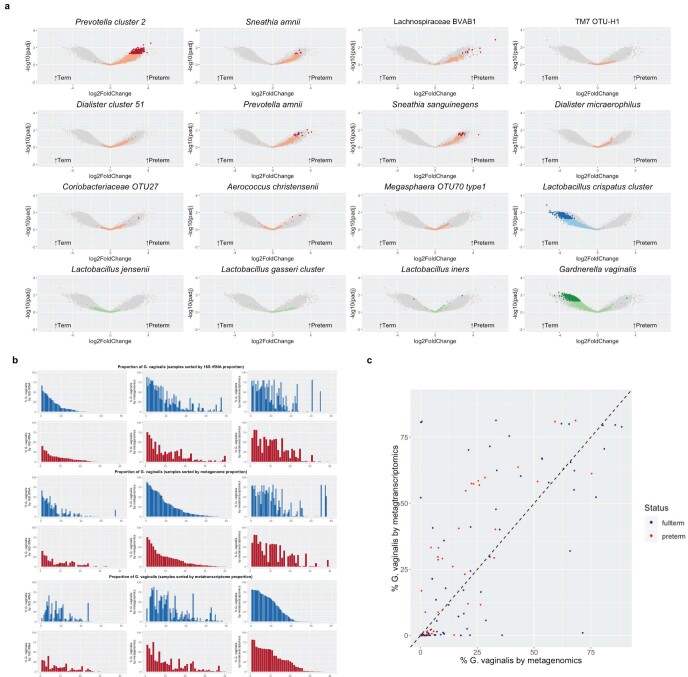
Genes differing in metagenomic and metatranscriptomic data between term and preterm cohorts. **a**, Volcano plot depicting genes that significantly differ between term (*n* = 41) and preterm (*n* = 81) cohorts in metagenomics data. Genes mapping to PTB-associated taxa, the *L. crispatus* cluster and four other common taxa that significantly differ at *P*_adj_ < 0.05 (two-sided) with FDR correction are shown in dark red, dark blue and dark green, respectively. **b**, Comparison of proportional abundance of *G. vaginalis* in preterm (red) and term (blue) vaginal samples as assayed by 16S rRNA (left), metagenomics (middle) and metatranscriptomics (right), sorted by 16S rRNA (top), metagenomics (middle) and metatranscriptomics (bottom) data. **c**, Scatter plot of the abundance of *G. vaginalis* by metagenomics (*x* axis) and abundance of *G. vaginalis* by metatranscriptomics (*y* axis) in pregnant women who delivered term (blue) or preterm (red). Two-sided Wilcoxon’s test of the ratio of the log-transformed proportions (log(metatranscriptomics *G. vaginalis* proportion)/log(*G. vaginalis* metagenomics proportion)) showed a significant difference between term and preterm groups (*P* = 0.01069).

Using the same approach with MGS data, we observed similar trends, but fewer genes were identified as statistically significant (*P*_adj_ < 0.05) overall (see Extended Data Fig. 7a and Supplementary Tables 9 and 10). Interestingly, 12.55% of the *G. vaginalis* genes analyzed using MGS data were significantly higher in the term cohort, whereas only one gene (that is, 0.02% of genes analyzed) that was identified as a hypothetical protein was higher (*P*_adj_ < 0.05) in the preterm cohort. We found the overall relative transcriptional rate of *G. vaginalis* was higher in preterm samples compared with term samples, using a calculated ratio of the proportion of reads mapped to genes in *G. vaginalis* reference genomes by MTS to reads mapped by MGS (Wilcoxon’s, *P* < 0.05; see Extended Data Fig. 7b,c). Previous studies have suggested that PTB risk differs with carriage of different *G. vaginalis* clades^25^. We extend this finding by showing data suggesting that PTB risk also differs with the transcriptional activity of *G. vaginalis*. Further investigations will be required to determine the underlying mechanisms that impact replication and transcription of different strains of *G. vaginalis* during pregnancy, and how these mechanisms affect women’s reproductive health and pregnancy.

**Extended Data Figure 8:**
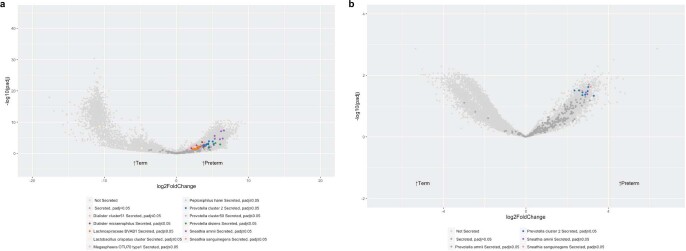
Genes predicted to encode secreted proteins or to be involved in bacterial secretion systems in metatranscriptomics and metagenomics datasets. Genes predicted to encode secreted proteins or to be involved in bacterial secretion systems that have very different transcript levels (**a**) or very different levels in metagenomics data (**b**) in term (*n* = 81) and preterm (*n* = 41) cohorts. Analysis was performed by mapping metatranscriptomics (**a**) or metagenomics (**b**) reads to a customized database of genomes representing 56 taxa. Comparative analysis was performed with DESeq2 using a global scaling approach. All genes with an FDR-adjusted *P*_adj_ < 0.05 (two-sided test), which are also predicted to be involved in bacterial secretion as identified by MacSyFinder, are shown and colored by taxon.

Genes encoding proteins involved in bacterial secretion systems play an important role in pathogenicity^53^. Thus, we examined all genes predicted to encode proteins involved in bacterial secretion in the database. We observed that 81% (71/91) of the predicted genes encoding secreted proteins that were far more transcriptionally abundant in the preterm cohort were from taxa identified as associated with PTB in our 16S rRNA analyses (see Extended Data Fig. 8a, Supplementary Tables 11 and 12, and Supplementary Data 4). The detected differences between the PTB and TB samples likely reflect the elevated proportional abundance of these taxa in PTB samples. Further studies are needed to determine whether these genes may contribute to mechanisms by which components of the vaginal microbiome may mediate or cause pathology or PTBs.

### Host cytokine expression in PTB

**Fig. 5:**
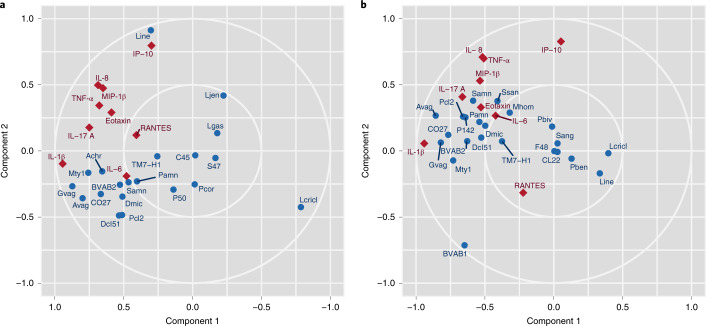
Sparse canonical correlation analysis. **a**,**b**, Cytokine abundance in vaginal samples from women who experienced TB (*n* = 90) (**a**) or PTB (*n* = 41) (**b**) were subjected to an integrative sCCA using log-transformed cytokine levels and log-transformed taxonomic profiling data (see  Methods). Blue circles represent bacterial taxa and red diamonds represent cytokines. Note that the component 1 axis for the TB sCCA (left) has been reversed for effective visual comparison with PTB sCCA. See Supplementary Table  3 for abbreviations. Note that, in sCCA analysis, factors (cytokines or microbial taxa) that are clustered tightly are highly correlated, and factors that are distant from each other are inversely correlated.

Of the nine cytokine levels examined in the present study (interleukin (IL)-1β, IL-6, IL-8, eotaxin, tumor necrosis factor (TNF)-α, IL-17A, macrophage inflammatory protein (MIP)-1β, interferon-γ-induced protein (IP)-10/chemokine ligand (CXCL)10, RANTES (regulated on activation, normal T cell expressed and secreted)), four (eotaxin, IL-1β, IL-6 and MIP-1β) were greatly increased in PTB relative to TB samples (false discovery rate (FDR)-adjusted *P* < 0.05 for each), consistent with previous reports of elevated IL-1, IL-6, MIP-1, IP10/CXCL10 and other proinflammatory cytokines associated with PTB in blood, amniotic fluid or cervical–vaginal lavage samples^54^. For further examination of the role of cytokines in the progression of pregnancy to PTB, we performed an integrative sparse canonical correlation analysis (sCCA) to assess the association of specific bacterial taxa with the abundance levels of nine key cytokines. For each participant, the sample corresponding to the earliest gestational age per trimester was characterized. In women who delivered at term (Fig. 5a), we observed a strong negative correlation between *L. crispatus* and several taxa associated with dysbiosis and PTB (for example, *G. vaginalis*, *Prevotella* cluster 2, *S. amnii* and, to a lesser extent, TM7-H1), as well as with the analyzed cytokines. The analyzed cytokines, which are largely proinflammatory, were loosely correlated both with each other and with taxa associated with dysbiosis and PTB. Notably, IP-10/CXCL10, which functions to induce chemotaxis of immune cells and promotes apoptosis, cell growth and angiostasis, and is generally considered to be proinflammatory^55^, was positively correlated with *L. iners*. This association was previously reported in the reproductive tracts of non-pregnant women from Kenya^56^. In contrast, in women who went on to experience PTB (Fig. 5b), the proinflammatory cytokines and dysbiotic taxa (for example*, A. vaginae, G. vaginalis* and *Megasphaera* type 1) formed a tighter cluster, indicating a stronger positive correlation, but IP-10/CXCL10 did not correlate with *L. iners*. Furthermore, BVAB1 was negatively correlated with IP-10/CXCL10 in these samples.

### Cross-study comparisons of the vaginal microbiome and preterm birth

**Extended Data Figure 9:**
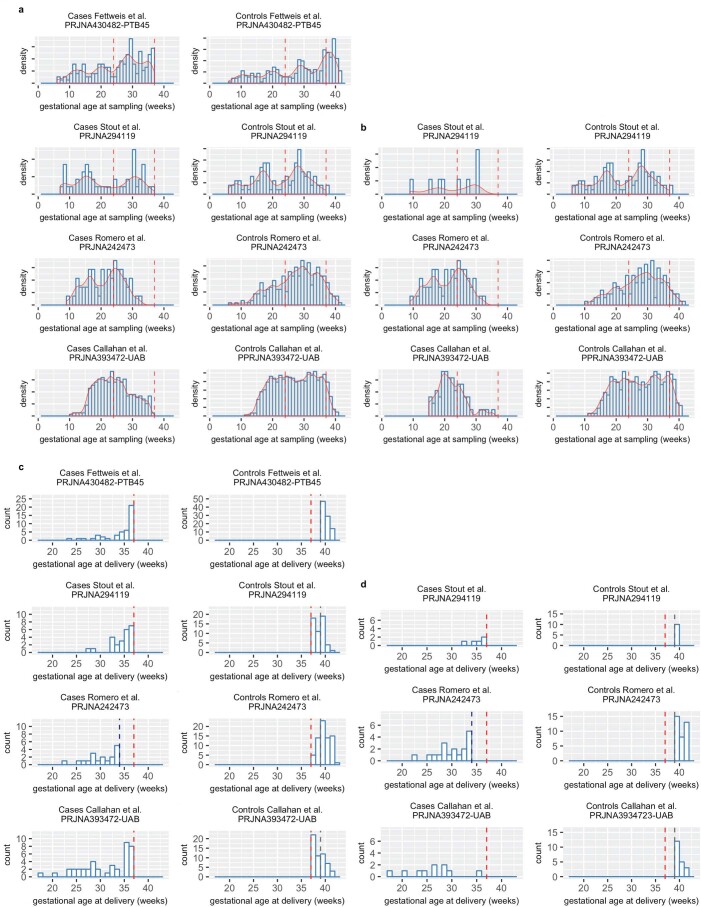
Distribution of sampling and gestational age at delivery across studies of the vaginal microbiome and PTB. The panels show the distribution of the gestational age at sampling (**a**) and gestational age at delivery (**c**) in preterm cases (left) and term controls (right) across four studies: the present study (top), the Stout et al. study, the Romero et al. study and the Callahan et al. UAB cohort (bottom), as originally published, and in **b**,**d** for the reanalyzed cohorts.

**Extended Data Figure 10:**
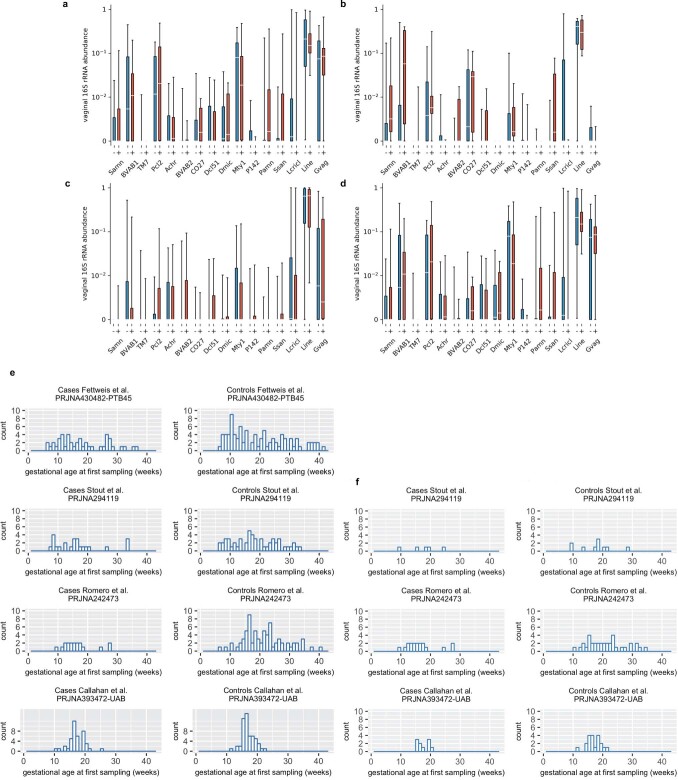
Proportional abundance shown as a log scale of candidate taxa identified in the present study across four PTB cohorts. **a**–**d**, Abundance values below 0.001 were rounded down to 0. Boxes show median and interquartile range; whiskers extend from minimum to maximum values within each cohort for the present study (PTB *n* = 45, TB *n* = 90) (**a**), the Stout et al. replication cohort (PTB *n* = 5, TB *n* = 10) (**b**), the Romero et al. cohort (PTB *n* = 18, TB *n* = 36) (**c**) and the Callahan et al. UAB replication cohort (PTB *n* = 10, TB *n* = 20) (**d**). The Mann–Whitney *U*-test (two-sided) for difference in proportional abundance between the PTB and TB cohorts, corrected using the Benjamini–Hochberg procedure with an FDR of 5% did not show statistical significance for these taxa in the cohorts shown in **b**–**d**. The earliest sample available for each subject was used (**f**). Earliest samples for the original four cohorts are shown in **e**.

Several recent studies^25,27,28,30^ generally reported limited correlation between the composition of the vaginal microbiome and PTB in cohorts of African descent. We compared the distributions of distinct candidate taxa for PTB risk across four studies^25,27,30^ of the vaginal microbiome in cohorts of pregnant women with predominantly African ancestry, including the MOMS-PI PTB study, with a harmonized reanalysis of the raw 16S rRNA sequencing reads (see Supplementary Tables 13 and 14, and Extended Data Figs. 9 and 10). There were non-trivial differences in every technical aspect of study design including sample collection, DNA extraction, PCR primers and conditions, sequencing platform, data quality and deposit which precluded an integrated analysis of these datasets (see Supplementary Table 13). Moreover, each of these studies varied markedly in cohort demographics, inclusion and exclusion criteria, and even the definition of PTB. PTB is understood to be a syndrome with many underlying causes^9^. Ascending infection of microbes from the vagina likely plays a causative role in some subtypes of PTB, but likely does not play a contributing role in all PTB. Thus, there were considerable differences (see Extended Supplementary Table 14) in the PTB case cohorts, as originally published in terms of the distribution of gestational age at delivery (see Extended Data Fig. 9), the percentage of women who had a non-medically indicated spontaneous PTB (37.5–100%), preterm premature rupture of the membranes (12.5–57.8%), treatment with progesterone (0–100%) or a history of PTB (29.1–100%). Our attempts to aggregate data from different studies highlight the opportunities for harmonization to obtain comparable data across studies.

We examined only the spontaneous preterm cases and we rematched controls 1:2 using the same approach from the present study to harmonize study designs across studies as far as possible for reanalysis. Sample sizes were small, in the range 5–18 spontaneous PTB cases, with controls matched 1:2. Although not statistically significant, likely due to sample size, cohort characteristics, and differences in experimental design and details as outlined above, we found partial support for candidate taxa identified in the present study (see Extended Data Fig. 10a–d). For example, the present study confirms an association between *Sneathia sanguinegens* and PTB, which was reported as significant (*P* < 0.05) before adjustment for multiple testing by Romero et al.^30^. Although there were only five spontaneous PTB cases reanalyzed in the Stout et al. cohort^27^, and only ten in the reanalyzed Callahan et al.^25^ cohort, we observed high concordance in the directionality of differences in abundance levels of preterm and term groups between these cohorts and the present study. We were also able to confirm that BVAB1, *Megasphaera* phylotype 1 and *Sneathia* species were elevated in a preterm cohort, which Nelson et al.^26^ previously reported as related to an increased risk for PTBs among women reporting a prior preterm delivery. The Nelson et al.^26^ study used quantitative PCR rather than 16S rRNA profiling, and the study was thus not included in the harmonized reanalysis. We also confirmed that *L. iners* and *G. vaginalis*, which were identified as vaginal microbial signatures associated with PTB in low-risk cohorts^25^, did not generalize to cohorts of African ancestry.

## Discussion

From a subset of 597 of the 1,572 pregnancies longitudinally sampled for the MOMS-PI study, we have generated omics data from more than 12,000 samples in one of the largest and most comprehensive multi-omic studies published to date. In addition, our analyses of longitudinal omics data from vaginal samples from 45 women who delivered preterm and 90 controls showed a signature of PTB in a cohort of women of predominantly African ancestry, including several taxa that have previously been implicated in adverse outcomes of pregnancy, including premature delivery^22,26,57–59^, in addition to taxa that have not been previously linked to adverse pregnancy outcomes. Women of African ancestry have a greatly increased risk of PTB compared with women of European ancestry^60^. Previous studies^16,21^ have shown that carriage of *L. crispatus*, which is negatively associated with PTB (see Fig. 2 and refs. ^22–25^), is more prevalent in women of European ancestry, and BVAB1, which is positively associated with PTBs, is more common in women of African ancestry. Thus, our findings are consistent with a proposed framework in which there is a spectrum of vaginal microbiome states linked to risk for PTB, and that these states vary across populations.

Our longitudinal modeling showed taxa associated with PTB tended to decrease in abundance in the vaginal environment throughout pregnancy, particularly in women of African ancestry. This finding is consistent with previous observations that pregnancy is associated with reduced carriage of bacterial vaginosis-associated organisms^21,27,57^. Considering that an adverse pregnancy outcome may be caused by ascension of pathogenic microbes, this trend suggests that the microbiome composition early in pregnancy may be most useful in the prediction of adverse outcomes. In a complementary analysis of an ethnically diverse cohort of women who delivered at term or early term in the MOMS-PI TB study, we show differences in the longitudinal dynamics of the microbiome in women of African ancestry compared with women of European ancestry^42^. In the present study, we developed a proof-of-concept model that suggests the presence of BVAB1, *Prevotella* cluster 2, *S. amnii* and TM7-H1 early in pregnancy may be useful for prediction of risk for PTB, particularly in high-risk populations. It is possible that BVAB1, *Prevotella* cluster 2, *S. amnii* and TM7-H1, and other taxa may have roles in the causation of PTB. As BVAB1 and TM7-H1 had not been cultivated or genetically characterized, we assembled their genomes from MGS data to search for clues to their pathogenic potential. We previously characterized the genome of *S. amnii*, identified potential cytotoxin genes and showed that cultured bacteria kill eukaryotic cells in vitro^61^. Although culture is not available for BVAB1 or TM7-H1, genomic factors identified in their genomes can now be genetically amplified and recombined into heterologous reporter systems and tested for pathogenic activity. Our MTS and MGS analyses supported the microbial signatures identified using 16S rRNA profiles.

Analysis of vaginal cytokine data from the MOMS-PI PTB study is consistent with previous findings showing that bacterial taxa generally associated with dysbiosis are highly correlated with expression of proinflammatory cytokines, which may play a role in the induction of labor. Labor is associated with proinflammatory cytokine expression, and premature labor can be induced by host inflammatory responses. We observed that vaginal IP-10/CXCL10 levels were inversely correlated with BVAB1 in PTB, inversely correlated with *L. crispatus* in TB and positively correlated with *L. iners* in TB, suggesting complex host–microbiome interactions in pregnancy.

Our findings contribute to an understanding of how microbial markers for PTB vary across populations. Vaginal microbiome composition as a whole and carriage rates of specific microbial taxa vary dramatically across populations, and thus it is not unexpected that the importance of relevant markers differs accordingly. Further studies are needed to determine whether the signatures of PTB reported in the present study replicate in other cohorts of women of African ancestry, to examine whether the observed differences in vaginal microbiome composition between women of different ancestries has a direct causal link to the ethnic and racial disparities in PTB rates, and to establish whether population-specific microbial markers can be ultimately integrated into a generalizable spectrum of vaginal microbiome states linked to the risk for PTB. Taken together, our data suggest that, coupled with other clinical and possibly genetic factors, microbiome-associated taxonomic, metabolic and immunologic biomarkers may be useful in defining the risk of PTB, and that this risk might be assessed early in pregnancy.

## Methods

### Participant enrollment, informed consent and health history collection

Participants for this study were enrolled from women visiting maternity clinics in Virginia and Washington State. All study procedures involving human subjects were reviewed and approved by the institutional review board at VCU (IRB no. HM15527). Participants were enrolled at multiple sites in Washington State by our partner registry, the GAPPS (see www.gapps.org) which was at the time under the umbrella of Seattle Children’s IRB (FWA00002443), IRB Application number 12879. Study protocols were harmonized across sites, and data and samples from participants enrolled in Washington State were distributed to the VCU site. Samples were collected from ten maternal body sites (that is, vaginal, cervical, buccal and rectal mucosa, blood, urine, chest, dominant palm, antecubital fossa and nares), five types of birth products (that is cord blood, amniotic fluid, placental membranes, placental tissue and umbilical cord) and seven infant body sites (that is, buccal and rectal mucosa, meconium/stool, chest, right palm, nares and respiratory secretions if intubated). All study participants enrolled in Virginia and most participants enrolled at Washington State sites were also enrolled in the RAMS Registry at VCU. RAMS Registry protocols were approved at VCU (IRB no. HM15528); GAPPS-associated sites ceded review to the VCU IRB through reliance agreements. Additional samples were collected from the participants enrolled in Washington State, which are archived at the GAPPS. The present study was performed with compliance with all relevant ethical regulations. Written informed consent was obtained from all participants and parental permission and assent were obtained for participating minors aged at least 15 years.

Pregnant women were provided literature on the project and invited to participate in the study. Women who (1) were incapable of understanding the informed consent or assent forms or (2) were incarcerated were excluded from the study. Comprehensive demographic, health history and dietary assessment surveys were administered, and relevant clinical data (for example, gestational age, height, weight, blood pressure, vaginal pH, diagnosis) were recorded. Relevant clinical information was also obtained from neonates at birth and discharge.

At subsequent prenatal visits, triage, in labor and delivery, and at discharge, additional surveys were administered, relevant clinical data were recorded and samples were collected. Vaginal and rectal samples were not collected at labor and delivery or at discharge. Women with any of the following conditions were excluded from sampling at a given visit:

1. Incapable of self-sampling due to mental, emotional or physical limitations
2. More than minimal vaginal bleeding as judged by the clinician
3. Ruptured membranes before 37 weeks
4. Active herpes lesions in the vulvovaginal region

### MOMS-PI PTB study case–control design

We initially selected 47 preterm cases of singleton, non-medically indicated PTBs from women who delivered between 23 weeks of gestation and 36 weeks and 6 days of gestation, and were selected from the 627 pregnancies in the Virginia arm of the study for whom gestational age at delivery was available at the time of the study design (phase 1 RAMS Registry cohort). From the phase 1 RAMS Registry cohort, 82 delivered before 37 weeks of gestation. Twelve of the participants who delivered preterm had multiple gestation pregnancies, twenty-one experienced medically indicated delivery, one delivered after fetal demise and one delivered a fetus at a non-viable gestational age. The participants had completed the study through delivery, and their gestational age information had been recorded in the study operational database at the time of the study design. We case matched the preterm participants 2:1 with participants who completed the study with singleton term deliveries ≥39 weeks, to avoid complications associated with early term birth^43–45^, with matching based on ethnicity, age and annual household income. With these criteria, we matched controls to cases as closely as possible, loosening criteria at each pass using an in-house script; a few difficult-to-match cases were matched by hand. Case matching was performed blinded to all other study data. Two of the 47 PTBs did not have 16S rRNA that passed quality control, so these PTB samples and their controls were excluded from the MOMS-PI PTB study.

### MOMS-PI TB study design

From an early subset of women who delivered in the MOMS-PI, we selected 90 pregnancies, including 41 women of European descent and 49 of African descent, who experienced term (≥39 weeks of gestation) or early term birth (between 37 weeks of gestation and 38 weeks and 6 days of gestation).

### Early pregnancy study design

An early pilot study was selected from a subset of 69 women in the MOMS-PI cohort from whom vaginal samples had been collected before 14 weeks of gestation. Targeted vaginal lipidomic profiles, vaginal cytokine profiles and vaginal 16S rRNA taxonomic profiles were generated.

### Maternal sampling schedule

Samples were collected from appropriately consented women at the enrollment visit, longitudinally at each prenatal visit, at triage, at labor and delivery, at discharge (~24–48 h after birth) and at postpartum follow-up visits. All swab samples were collected with BD BBL CultureSwab EZ swabs. Self-sampling has been shown to provide samples equivalent to those collected by a trained clinician. Vaginal and rectal samples were collected either by healthcare providers during a pelvic exam (no speculum) or by self-sampling longitudinally throughout pregnancy and at postpartum follow-up visits. Vaginal and rectal samples were not collected at the discharge visit. Research coordinators instructed the participants on self-sampling procedures, provided a self-sampling instructional brochure and provided the participant a room for self-sampling. Maternal buccal samples were collected by a research coordinator during a study visit or by self-sampling at all visit types. Blood samples were collected at select visits by phlebotomists. Urine samples were longitudinally collected using a clean-catch protocol for a subset of participants. Cervical samples were collected by a clinician during a pelvic exam using a speculum at select visits. Samples from the antecubital fossa and nares were collected by research coordinators longitudinally throughout pregnancy, at discharge visits and at postpartum follow-up visits, but not at triage visits. At discharge visits, dominant palm and chest (skin) samples were also collected by a research coordinator. For samples collected at RAMS Registry sites, the following birth products were also collected: placental membranes, placental tissue, umbilical cord, cord blood and amniotic fluid (cesarean section only). Birth products, urine and additional blood samples for the participants enrolled through GAPPS sites are archived at the GAPPS Repository.

### Infant sampling schedule

Samples from neonates (for example, buccal, rectal, meconium/stool, right palm, chest and nares) were collected at birth (~1 h after birth) and at discharge (~24–48 h after birth). For infants admitted to the neonatal intensive care unit (NICU), additional samples were collected at days 3 and 7 of life and weekly until discharge from the NICU, or until the infant’s first birthday. In the NICU, respiratory secretions were also collected from infants who were intubated. Buccal, rectal, right palm, chest and nares swabs were collected by healthcare providers. Meconium/stool samples were collected by research coordinators from diapers using sterile CultureSwab EZ swabs.

### Swab sample preprocessing

Swab samples were collected as follows: (1) maternal mid-vaginal wall: a double-tipped CultureSwab EZ swab was inserted ~5 cm into the vagina, pressed against the vaginal sidewall, rotated for 5 s and removed; (2) maternal cervical: during a speculum exam, a single-tipped CultureSwab EZ swab was inserted into the endocervix to the depth of the entire tip of the swab, rotated 360°, held for 10 s and removed, being careful not to contact the vaginal walls; (3) maternal and infant buccal: a double-tipped CultureSwab EZ was placed firmly in the mid-portion of the cheek, rotated for 5 s and removed; (4) maternal rectal: a double-tipped CultureSwab EZ swab was inserted to a depth of ~2.5 cm into the rectum, rotated for 5 s and removed; (5) infant rectal: a single-tipped CultureSwab EZ swab was inserted to a depth of up to ~0.64 cm into the rectum, rotated for 5 s and removed; maternal and infant nares samples were collected using single-tip CultureSwab EZ swab dipped in sterile saline and inserted ~1.27 cm for maternal samples (up to ~0.64 cm for infant) in the left nostril, rotated for 5 s and removed; (6) maternal and infant chest samples were collected using a double-tipped CultureSwab EZ swab dipped in sterile saline, pressed against the chest and rotated for 5 s; maternal dominant palm and infant right palm samples were collected; (7) maternal dominant palm and infant right palm samples were collected using a double-tipped CultureSwab EZ swab dipped in sterile saline, pressed against the palm and rotated for 5 s; (8) vaginal pH was collected using commercial applicators with pH paper. Briefly, the applicators were inserted ~3.8–5 cm into the vagina, applied gently to the vaginal wall and withdrawn. The research coordinator compared the color of the pH indicator with a color chart and recorded the vaginal pH.

Swabs were preprocessed and stored at −80 °C within an hour of collection. Material from swabs was transferred to the solutions compatible with downstream omics assays by vigorous spinning of swabs against the side walls of tubes for 15 s. Maternal vaginal, maternal and infant buccal, maternal and infant rectal, maternal cervical, maternal and infant chest, maternal dominant palm, infant right palm and maternal antecubital fossa swabs for DNA isolation were immersed in 750 μl MoBio PowerSoil DNA Isolation buffer; maternal vaginal, maternal and infant buccal, maternal and infant rectal, maternal cervical, maternal and infant chest, maternal dominant palm, infant right palm and maternal antecubital fossa swabs collected to be preserved for cultivation studies were immersed in 1 ml of culture medium (brain heart infusion supplemented with 1% yeast extract, 2% gelatin, 0.1% starch, 1% glucose and 20% glycerol); maternal vaginal swabs for RNA purification were immersed in RNAlater (Qiagen); maternal vaginal and maternal and infant buccal swabs for cytokine profiling were immersed in 500 μl of 10 mM Tris, pH 7.0, 1 mM ethylenediaminetetraacetic acid; maternal vaginal and maternal and infant buccal swabs were also immersed in 250 μl of 10 mM Tris, pH 7.0, 1 mM ethylenediaminetetraacetic acid; and maternal vaginal swabs were immersed in 500 μl of 0.01% butylhydroxytoluene phosphate-buffered saline solution for lipid analyses.

### Sample processing

DNA purification was performed using the MoBio PowerSoil Kit, as described by the manufacturer. RNA purification was performed using the MoBio PowerMicrobiome RNA Isolation Kit as described by the manufacturer. Total RNA was depleted of human and microbial rRNA using the Epicentre/Illumina Ribo-Zero Magnetic Epidemiology Kit, as described by the manufacturer. DNA and RNA samples were stored at −80 °C.

### 16S rRNA taxonomic surveys of the vaginal microbiome

DNA in each sample was amplified with barcoded primers targeting the V1–V3 region of the 16S rRNA and validated for vaginal taxa as previously reported^47^. Primer sequences are listed in Supplementary Table 8. The samples were randomized at the PCR stage and again at the sequencing stage. Samples were multiplexed (384 samples per run) and sequenced on our Illumina MiSeq sequencer using 600 cycles creating 2 × 300 bp paired-end reads to generate a depth of coverage of at least 50,000 reads per sample. The raw sequence data were demultiplexed into sample paired-end fastq files based on unique barcode sequences using a customized Python script. The preprocessing of sequences was performed using the MeFiT^62^ pipeline, with amplicons (on average ~540 base-pairs (bp) long) generated by merging the overlapping tails of paired-end sequences, followed by quality filtering using a MEEP (maximum expected error rate) cutoff of 1.0. Data were processed using harmonized bioinformatics pipelines with the other iHMP projects for upload to the Human Microbiome Project Data Coordination Center (HMP DACC). Non-overlapping, high-quality reads were assigned to operational taxonomic units (OTUs), using the reference-based clustering method implemented by the *pick_closed_reference_otus.py* script in the QIIME package. For data uploaded to the HMP DACC, the reference database used is the subset of Greengenes 16S rRNA sequence database clustered at 97% identity.

For each sample, the raw paired-end reads were uploaded to the HMP DACC and NIH Sequence Read Archive (SRA) as FASTQ files (*16SRawSeqSet* node), the quality-filtered reads as FASTQ files (*16STrimmedSeqSet* node) and the qiime output OTU tables in the biom format (*16s_community)*.

For analysis of vaginal samples in the MOMS-PI PTB cohort, an alternate bioinformatics method was used. Non-overlapping, high-quality reads were screened for chimeric sequences with UCHIME^63^ against our custom database of vaginally relevant taxa. Each processed 16S rRNA gene sequence was taxonomically classified to the species level using STIRRUPS^47^, which aligns against a custom reference database using USEARCH. Reference sequences for *Prevotella* cluster 2 include *P. buccalis*, *P. timonensis*, *Prevotella* OTU46 and *Prevotella* OTU47. Only samples with at least 1,000 reads that met filtering criteria were analyzed. For vaginal samples, the STIRRUPS output tables were also uploaded to the HMP DACC (*16s_community)*.

### Whole shotgun metagenomic/metatranscriptomic sequencing

DNA libraries were prepared using KAPA Biosystems HyperPlus Library Kit and sequenced on our Illumina HiSeq 4000 (2 × 150 b PE). We sequenced all available vaginal samples for the PTB and TB cohorts, multiplexed 24 samples per lane and obtained ~1–2 × 10^7^ 150-nucleotide reads per sample. For MGS, we subjected 555 vaginal samples to sequencing and 542/555 (97.7%) had a minimum of 100,000 post-quality control (QC)-filtered read pairs. For MTS, we selected samples in the second trimester and the last collected sample for sequencing. If samples were not available in the second trimester, samples before and after the targeted second trimester window were selected. RNA was extracted from a total of 337 samples, of which 243 (72.10%) had at least 10 ng μl^–1^ of RNA and were submitted for sequencing. Of these 242/243 (99.6%) met our minimum of 100,000 post-QC-filtered read pairs. The rRNA-depleted messenger RNA was prepared for sequencing by constructing complementary DNA libraries using the KAPA Biosystems KAPA RNA HyperPrep Kit. Indexed complementary DNA libraries were pooled in equimolar amounts and sequenced on the Illumina HiSeq 4000 instrument, running four multiplexed samples per lane, with an average yield of ~100 Gb per lane, sufficient to provide >100× coverage of the expression profiles of the most abundant 15–20 taxa in a sample.

### Whole shotgun metagenomic/metatranscriptomic data pre-processing

Raw sequence data were demultiplexed into sample-specific fastq files using *bcl2fastq* conversion software from Illumina. Adapter residues were trimmed from both the 5′- and the 3′-end of the reads using Adapter Removal tool v.2.1.3. The sequences were trimmed for quality using MEEPTOOLS^64^, retaining reads with a minimum read length of 70 b and MEEP quality score <1. Human reads were identified and removed from each sample by aligning the reads to the hg19 build of the human genome, using the BWA aligner. Taxonomic classification and relative abundance of bacteria in the metagenomes and metatranscriptomes, using harmonized bioinformatics pipelines with the other iHMP projects for upload to the HMP DACC, were obtained using Metaphlan2 (ref. ^65^), with default parameters. The human-filtered MGS and MTS nodes were created at HMP DACC, *WGSRawSeqSet* and *MicrobTranscriptomicsRawSeqSet*, respectively, which link to the controlled-access data at the database of Genotypes and Phenotypes (study no. 20280). Metaphlan2 output community profiles of metagenomes and metatranscriptomes have been uploaded to HMP DACC, *wgs_community* node and *microb_metatranscriptome* node, as tab-delimited text files.

### Functional analysis of metagenomic and metatranscriptomic sequencing reads

For vaginal samples in the MOMS-PI PTB study, assignment of MGS and MTS reads to known genes/pathways was performed using ASGARD^66^, HUMAnN2 (ref. ^67^) and ShortBRED^68^. The reads were also compared with appropriate databases (KEGG, GO, COG, etc.) using BLAST or other alignment tools to characterize functional data about these samples. HUMAnN2 output functional profiles of metagenomes and metatranscriptomes have been uploaded to HMP DACC, *wgs_functional* node and *microb_metatranscriptome* node, as tab-delimited text files.

### BVAB1 genome assembly from metagenomic reads

Starting with high-quality, trimmed, MGS reads from one sample with a high abundance of BVAB1, human reads were removed by alignment to the human hg19 reference genome using BWA alignment software. Human-filtered reads were digitally normalized with BBMap (https://sourceforge.net/projects/bbmap) with a target coverage of >40× to remove reads from highly repetitive elements of the genomes that may hamper the de novo assembly process, and to ensure that reads originating from PCR duplication were excluded before assembly. Reads were assembled with SPAdes v.3.8.0 using the ‘-meta’ option to generate a consensus assembly scaffold. Before clustering the scaffolds generated by SPAdes v.3.8.0, the human depleted reads were aligned back to the scaffolds using Bowtie2 with the ‘--very-sensitive’ option for global alignment. The resulting bam files were converted into ‘scaffold-to-average coverage’ maps using a customized Python script. These contigs were clustered into individual genomes using MyCC^69^, with tetramer frequencies coupled with the average coverage. Assembly identity was confirmed by alignment with 16S rRNA sequences from BVAB1. Reads were mapped back to individual MyCC clusters and then submitted to a new assembly using Newbler Assembler v.2.8. Where necessary, gaps were closed by sequencing of PCR amplicons using primers directed to contig ends. Coverage of the final genome averaged over 40× and completion was confirmed by the presence of all 40 highly conserved marker genes commonly used to assess genome assemblies^69^. Genome sequences were annotated with in-house pipelines using Prokka and ASGARD^66^.

### TM7-H1 genome assembly from metagenomic reads using PacBio

DNA from a sample with high proportional abundance of TM7-H1 was sent to Pacific Biosciences for PacBio sequencing, using the TdT protocol, which is suitable for sequencing low-input samples. An HGAP metagenome assembly was performed using a white list to exclude reads mapped to human, which yielded three TM7-H1 contigs. Genome sequences were annotated with in-house pipelines using Prokka and ASGARD^66^.

### Mapping of metagenomic and metatatranscriptomic sequencing reads to a customized vaginal genome database representing 56 STIRRUPS taxa

We curated a custom database of vaginal genomes for 56 taxa identified in the 16S rRNA dataset that had a 0.1% average proportional abundance or for which at least 5% of samples were present at 0.1%. We also included reference genomes for *Chlamydia trachomatis* and *Neisseria gonorrhoeae*. Reference genomes are not available for several STIRRUPS taxa that met one or both of these criteria: *Bacteroides coagulans*, Clostridiales BVAB2, *Dialister* cluster 51, *Dialister propionicifaciens*, Lachnospiraceae OTU33, Prevotellaceae OTU61 and Proteobacteria OTU-T1. An average of 95.2% of MGS reads from samples in the full-term cohort and 94.3% of MGS reads from samples in the preterm cohort were identified as human (see Extended Data Fig. 4). A significantly smaller percentage of MTS reads (that is, an average of 30.0% and 32.8% in the full-term and preterm cohorts, respectively) were identified as human. This likely reflects the sampling of dead or dying vaginal epithelial cells. Bowtie2 default parameters were used to map filtered, non-human-read MTS and MGS reads to the customized vaginal genome database. All genomes were reannotated using Prokka. MacSysFinder was used to identify genes involved in bacterial secretion systems in the genomes in the reference database^70^. FeatureCounts was used to count paired-end reads where both ends mapped to non-ribosomal genes (coding sequences, transfer RNAs and transfer-messenger RNAs). Ribosomal genes were not included because we were interested in testing candidate taxa using other genes to build confidence that identification of candidate taxa was not dependent on 16S rRNA microbiome-profiling protocol choices. Ribosomal genes were thus classified as unassigned, no features. Chimeras were excluded, only primary alignments were mapped and duplicate reads were excluded. Paired MGS and MTS data were available for 41 women who delivered preterm and 81 term controls.

To confirm that the microbial signatures of PTB identified using 16S rRNA data were not attributable to protocol choices or protocol biases, we used MGS and MTS data to support these findings. The MGS data and 16S rRNA profiles were typically generated from the same DNA preparations, but they used independent sequencing strategies and bioinformatics and analysis methods. The MTS data were generated from vaginal swab samples collected in parallel with those used for DNA extractions, but the downstream protocol was entirely different. We analyzed one time point per participant, with a mean gestational age of sampling at 25 weeks for the preterm cohort and 26 weeks for the full-term cohort, and used a global scaling approach to normalize to all genes in the 56 taxa in our database. DESeq2 (ref. ^71^) was used to compare term and preterm cohorts using an organism-independent, global-scaling approach; genes with fewer than 1,000 total mapped genes across samples were excluded from analysis. Note that, with global scaling, it is not possible to differentiate differences caused by differential abundance and those due to differential expression in MTS data. Thus, read counts mapping to a bacterial gene from a specific taxon were normalized to the total number of reads mapping to all bacterial genes in the database.

Given that our MTS results reflect findings from 16S rRNA microbiome analyses, the results are most likely largely driven by differential abundance between term and preterm cohorts. For comparative analyses across 16S rRNA, MGS and MTS, the proportional abundance was calculated based on the 56 taxa that could be measured across all three approaches. To calculate the taxonomic proportional abundance for an MGS or MTS sample, the sum of reads mapped to genes for a given taxon was divided by the total number of reads mapped to all bacterial genes in the database. For this analysis, the 16S rRNA counts for a sample were renormalized by dividing the total number of reads mapped to a taxon by the total reads mapped to all taxa represented in the custom genome database, to which the MGS and MTS samples were mapped.

### Metabolic modeling

Draft constraint-based metabolic models^72^ for TM7-H1, BVAB1 and *L. crispatus* were generated using functional annotation information with Enzyme Commission numbers to describe function and KEGG IDs for nomenclature. Model contents are provided in Supplementary Information File 6 as .xls files and mapping files are provided as .xml files. Draft models have not been gap filled, given that most of these organisms are poorly characterized, and previously studied species of TM7 have been found to be non-free living.

### MOMS-PI PTB study: cytokine profiling

The Bio-Plex Pro Human Cytokine 27-plex Assay panel (M50-0KCAF0Y, Bio-Rad) was used to measure cytokine concentrations according to the manufacturer’s protocol. Briefly the frozen vaginal swab samples were thawed on ice and centrifuged at 10,000*g* for 10 min at 4 °C and diluted fourfold in 100 mM Tris buffer, pH 7.5. The assay was carried out on a black 96-well plate (10021013, Bio-Rad), and 50 µl of cytokine standard, interassay QC (described below) and sample were added in duplicate to appropriate wells. The Bio-Plex MAGPIX Multiplex Reader was used for data acquisition with default settings. Bio-Plex Manager v.6.0 software was used for data analysis using five-parameter logistic (5-PL), non-linear regression model on optimization for all analytes within 70–130% of the recovery range.

The interassay QC control was prepared from lipopolysaccharide-stimulated cell culture medium. Briefly, VK2/E6E7 (American Type Culture Collection (ATCC) CRL-2616) cells were initially grown in T75 flasks in Dulbecco’s modified Eagle’s medium/F-12 supplemented with 10% FBS (11320–033, 26140079, ThermoFisher) at 37 °C, 5% CO_2_ to confluency. These cells were trypsinized and reseeded at a concentration of 3 × 10^5^ cells ml^–1^ per well on a 24-well plate (82050–892, VWR). After 24 h, the medium was replaced with the fresh medium containing 100 ng ml^–1^ lipopolysaccharide (L2630-10MG, Sigma). Twenty-four hours post-lipopolysaccharide treatment, the cell culture medium was harvested, pooled and centrifuged at 3,000*g*  for 10 min at 4 °C. The resultant soluble fraction was aliquoted and stored at −80 °C for use as assay QC. Out-of-range cytokine concentration values were imputed with the upper or lower limit of detection for the specific cytokine where necessary. Nine cytokines (that is, IL-1β, eotaxin, IL-8, TNF-α, IL-17A, MIP-1β, IL-6, IP-10/CXCL10 and RANTES) had fewer than 30% out-of-range values and were selected for analysis. Cytokine concentrations normalized per swab sample were uploaded to HMP DACC (*host_cytokine* node) as tab-delimited text files. Cytokine data from the MOMS-PI PTB study, the MOMS-PI TB study and the MOMS-PI Early Pregnancy study were not designed to be used in concert due to differences in lots of reagents and normalization protocols. Sub-study tags have been uploaded to the HMP DACC to discriminate samples by study.

### MOMS-PI TB study: cytokine profiling

The Bio-Rad Bio-Plex Pro Human Cytokine 27-Plex Assay was employed with a Luminex 100/200 System to quantify cytokine levels in the vaginal samples. Frozen vaginal swab samples suspended in 500 μl 100 mM Tris-HCl, pH 7.5, were thawed on ice and centrifuged at 10,000*g* for 10 min at 4 °C. The Bio-Plex assay was conducted on samples and serial dilutions of standards in duplicate, according to the manufacturer’s instructions. Values were analyzed using a 5-PL, non-linear regression curve model. Cytokine values deemed out of range were assigned the upper or lower limit of detection for the specific cytokine. Cytokine concentrations were uploaded to the HMP DACC (*host_cytokine* node) as tab-delimited text files. The values at the DACC were not normalized to protein concentration as determined by the Bradford Assay^39^. Cytokine data from the MOMS-PI PTB study, the MOMS-PI TB study and the MOMS-PI Early Pregnancy study were not designed to be used in concert due to differences in lots of reagents and normalization protocols. Sub-study tags have been uploaded to the DACC to discriminate samples by study.

### MOMS-PI Early Pregnancy study: cytokine profiling

The Bio-Rad Bio-Plex Pro Human Cytokine 27-Plex Assay was employed with a Luminex 100/200 System to quantify cytokine levels in the vaginal samples. Frozen vaginal swab samples suspended in 500 μl 100 mM Tris-HCl, pH 7.5, were thawed on ice and centrifuged at 10,000*g* for 10 min at 4 °C. The Bio-Plex assay was conducted on samples and serial dilutions of standards in duplicate, according to the manufacturer’s instructions. Values were analyzed using a 5-PL, non-linear regression curve model. Cytokine values deemed out of range were assigned the upper or lower limit of detection for the specific cytokine. IL-2, IL-5 and IL-15 levels were below the limit of detection in >50% vaginal samples and were not included in subsequent analyses. Although biological replicates could not be performed due to the effects of multiple freeze–thaw cycles on sample viability, the intra-assay coefficient of variation was found to be acceptable at <7% for all cytokines. Cytokine concentration (pg ml^–1^) was divided by total protein concentration (mg ml^–1^) to yield normalized cytokine concentration (pg cytokine mg protein^–1^) for each sample. Samples for which total protein could not be determined were not included in the analysis. Cytokine concentrations were uploaded to the HMP DACC (*host_cytokine* node) as tab-delimited text files. The values at the DACC were not normalized to protein concentration as determined by the Bradford Assay^39^. Cytokine data from the MOMS-PI PTB study, the MOMS-PI TB study and the MOMS-PI Early Pregnancy study were not designed to be used in concert due to differences in lots of reagents and normalization protocols. Sub-study tags have been uploaded to the DACC to discriminate samples by study.

### Early Pregnancy study: lipidome profiling of vaginal samples

A pilot study was performed for lipidome profiling of vaginal samples. For eicosanoid and sphingolipid quantification, an equal volume of ethanol containing 10 ng of eicosanoid internal standards or methanol containing 50 pmol of sphingolipid internal standards was added to clarified vaginal swab contents dispersed in phosphate-buffered saline containing 0.01% butylhydroxytoluene. Eicosanoid internal standards consisted of 30 deuterated analytes, including (*d*_4_) 6-keto-prostaglandin (PG)F-1α, (*d*_4_) PGF-2α, (*d*_4_) PGE-2, (*d*_4_) PGD-2, (*d*_4_) LTB_4_ (leukotriene B_4_), (*d*_4_) thromboxane (Tx)B_2_, (*d*_4_) LTC_4_, (*d*_5_) LTD_4_, (*d*_5_) LTE_4_, (*d*_8_) 5-hydroxyeicosatetranoic acid, (*d*_8_) 15-hydroxyeicosatetranoic acid, (*d*_8_) 14,15-epoxyeicosatrienoic acid, (*d*_8_) arachidonic acid and (*d*_5_) eicosapentaenoic acid. Sphingolipid internal standards consisted of *d*_17_ sphingosine, sphinganine, sphingosine-1-phosphate, sphinganine-1-phosphate and *d*_18:1/12:0_ ceramide-1-phosphate, sphingomyelin, ceramide and monohexosylceramide (Avanti). After centrifugation at 12,000*g* for 20 min, the resultant mixture was subjected to UPLC electrospray ionization mass spectrometry/mass spectrometry analysis using a hybrid, triple quadrupole, linear ion trap mass analyzer (ABSCIEX 6500 QTRAP) via multiple-reaction monitoring. Detailed separation, elution and ionization conditions have been previously described^73^ and are summarized in the Supplementary Appendix 1. Spectral data were analyzed using MultiQuant software (ABSCIEX) and quantification was carried out by comparison against known quantities of internal standards and a seven-point dilution curve. The values were uploaded as tab-delimited text files to the HMP DACC. The values at the DACC were not normalized to protein concentration as determined by the Bradford Assay^39^. Cytokine data from the MOMS-PI PTB study, the MOMS-PI TB study and the MOMS-PI Early Pregnancy study were not designed to be used in concert due to differences in lots of reagents and normalization protocols. Sub-study tags have been uploaded to the DACC to discriminate samples by study.

### Community state types/vagitypes

Vaginal 16S rRNA profiles were assigned to community state types (CSTs) based on the taxon with the largest proportion of reads. Samples in which the largest proportion was less than 30% were not assigned a CST/vagitype. This ‘predominant taxon’ rule has been shown to exhibit over 90% agreement with clustering-based methods across a variety of vaginal microbiome datasets^46^, and yet is not population or dataset dependent and is therefore more conducive to use in a clinical setting. Differences in the numbers of *L. crispatus* CSTs among the PTB and TB cohorts were tested using a Fisher’s exact test.

### Markov chain analysis

The R package *msm* was used to fit a continuous-time Markov chain model for CST transitions. The model takes as input the subject, CST/vagitype and gestational age in days for each sample. The states were *L. crispatus, L. iners*, BVAB1*, G. vaginalis* and ‘Other’. The pregnancy outcome (that is, preterm or term birth) was included as a covariate. To derive confidence intervals for maximum likelihood estimates of transition probabilities, the set of transitions modeled was restricted to those in which at least four transitions were observed between subsequent visits for individuals in the study. Without this restriction, the maximum likelihood method does not converge. A stationary distribution for each group was estimated by setting the time to 100,000 days. Dynamic balance was checked for the one-trimester transition probabilities by taking the difference between the forward and reverse transition probabilities.

### Filtering out low-abundant taxa

As the first step in analyzing each dataset of vaginal 16S rRNA profiles, we analyzed the abundance of each taxa present in the profiles, and removed from further consideration low-abundant species. We used two abundance criteria: we retained taxa that either (1) 5% of the profiles exhibited an abundance of at least 1%, or (2) at least 15% of profiles exhibited an abundance of at least 0.1%. Taxa that failed to meet both (1) and (2) were removed.

### Univariate analysis to identify taxa very different in abundance in PTB and TB cohorts

We analyzed vaginal 16S rRNA profiles from 135 participants, 45 who delivered preterm and 90 who delivered term. The microbiome profile of the earliest sample from each of these women was used in this analysis. In this dataset, 26 taxa remained after filtering out the low-abundance taxa. For each of these 26 most abundant taxa, we performed a Mann–Whitney *U*-test to identify important differences in the presence and abundance in PTB and TB cohorts. For this analysis, abundance values below 0.00001 were rounded to zero. Taxa abundance was considered greatly different between cohorts if the *q* value was less than an FDR of 5% after correction via the Benjamini–Hochberg procedure. For each taxon we also calculated the median and the 75th percentile in the PTB and TB cohorts.

### Longitudinal models

A GAMM^74^ incorporating BMI, ethnicity (African, European), pregnancy outcome (preterm, full term), a smoother for gestational age and a random subject effect was used to longitudinally model log-transformed relative abundances of vaginally relevant taxa. Effect contributions were determined using analysis of variance tests. The model is described by the formula:

$${\mathrm{log}}\left( {{\mathrm{Abundance}}} \right)_{ij} = {\mathrm{\beta }}_0 + {\mathrm{\beta }}_1{\mathrm{PO}}_I + {\mathrm{\beta }}_2{\mathrm{BMI}}_{ij} + {\mathrm{\beta }}_3{\mathrm{pH}}_{ij} + {\mathrm{\beta }}_4I_{\mathrm{{ethnicity}}} + f\left( {{\mathrm{ga}}_{ij}} \right) \times {\mathrm{PO}}_i + \gamma _I + \varepsilon _{ij}$$

where log(Abundance)_*ij*_ is the log-transformed taxa relative abundance for the *j*th observation of the *i*th subject, PO is pregnancy outcome (preterm, full term), pH is vaginal pH, *I*_ethnicity_ is an indicator variable that takes on the value 0 for subjects of African ancestry and 1 for subjects of all other ancestries, ga is gestational age, *f*(.) is a smooth function, *γ*_*i*_ is the random effect for the *i*th subject and *ε*_*ij*_ is the error term.

The degree of smoothness for gestational age was estimated by restricted maximum likelihood^75^. Models were fit using the gamm4: GAMMs using mgcv and lme4 package in R, released by Wood and Scheipl in 2017.

### Canonical correlation analysis of cytokines and vaginal microbiome profiles

An integrative analysis of both log-transformed 16S rRNA survey data and log-transformed cytokine data was performed using sCCA^76^. Classic canonical correlation analysis^77^ explores the correlation between two sets of quantitative variables measured on the same subjects. The sCCA introduces an l1-penalization term to handle the case of more variables than observations. Nine cytokines, with fewer than 30% out-of-range values, were selected for analysis (that is, IL-1β, eotaxin, IL-8, TNF-α, IL-17A, MIP-1β, IL-6, IP-10/CXCL10, RANTES). Out-of-range cytokine concentration values were then imputed with the upper or lower limit of detection for the specific cytokine where appropriate. For each subject, the observation corresponding to the earliest gestational age per trimester was used for analysis. We performed sCCA separately for full-term and preterm subjects using the sgcca function in the R package mixOmics^78^.

The results of sCCA are displayed in a correlation circle plot^77^. The coordinates of the plotted points (variables) are the correlations between the variables and their canonical variates. Variables that have a strong positive correlation are projected close to each other on the plot, whereas variables that are negatively correlated are plotted opposite each other. The greater the distance from the origin, the stronger the relationship among variables^77^. The correlation circle plots are constructed using the plotVar function in the R package mixOmics.

### Taxon co-occurrence

Bacterial taxa were determined to be present if they comprised ≥0.1% of the total vaginal microbiome profile. We utilized the statistical tool REBACCA^79^ to mitigate the effects of relative constraint. REBACCA was run using 50 bootstraps and a visualization of bacterial correlations was generated using Gephi. Correlations with more than 0.3 or less than −0.3 are shown, with negative correlations in red and positive correlations in blue. Edge weights are representative of the strength of correlation between taxa and the four major predictive taxa, shown in gray.

### Predictive modeling of PTB using early pregnancy microbiome profiles

We constructed a linear predictive model of PTB as follows: from the full cohort, we selected subjects who had at least one vaginal 16S rRNA sample early in the pregnancy, in days 42–167 (inclusive) of gestation. A total of 31 PTB and 59 TB subjects had at least one sample in this time window; if multiple samples were present in that window, we used the earliest sample.

We first filtered out low-abundant species in this dataset: 25 passed the selection criteria. For these taxa, the abundance data were soft-thresholded with a 0.001 threshold, to reduce the impact of statistical noise resulting from low-abundance values, by subtracting 0.001 from the abundance and setting all resulting negative values to 0, and log-transformed through a transform log_10_((abundance + 0.001)/0.001), where dividing by 0.001 shifts the logarithm values for abundances in the zero (0.0) to 1 (1.0) range from negative to non-negative values. Ten taxa were significantly different between the PTB and TB cohorts.

The model construction uses a two-step procedure: first, we applied a Mann–Whitney *U*-test to all species that survived the abundance-based filtering criteria, retaining species with a two-sided *P* value of 0.05 or less. Based on these species, the predictive model was trained using logistic regression with L_1_ regularization^80^, to reduce the impact of collinearity between species and the resulting sign reversals and false detections. Regularized logistic regression finds a vector of taxa weights **w** that minimizes: Σ_*i*_ln(1 + ${e^{(-y_i\,w^Tx_i)}}$) + *C* ||**w**||_1_ over the training set of samples (***x***_***i***_, *y*_*i*_). The constant C was selected based only on samples from the training set, using grid search and nested cross-validation.

The statistical significance of the model in the form of a *P* value was estimated using a permutation test, consisting of training 10,000 models on data with the class variable randomly permuted before processing, and comparing the distribution of the 10,000 AUROC values with the AUROC values of the original model trained using unperturbed class variable. Performance of the model on previously unseen samples was assessed using the leave-one-out method. We assessed sensitivity, specificity and AUROC, as implemented in the Python scikit-learn package^81^.

The PTB predictive score was defined as: 0.775 log_10_(Samn) + 0.751 log_10_(BVAB1) + 0.116 log_10_(TM7) + 0.011 log_10_(Pcl2). To avoid negative values of the logarithm for abundance data on the [0,1] scale, we used the logarithm in the form log_10_((abundance + 0.001)/0.001), where abundance is soft-thresholded at 0.001, that is, 0.001 is subtracted from abundance values, and negative values are replaced by 0. This transformation shifts log(abundance) from the negative [−3,0] range to the non-negative [0–3] range, but does not affect the relative positions of the samples.

### Predictive modeling of PTB using clinical variables

For comparison with the microbiome-based predictive model, we also trained a predictive model using only clinical variables, without any input from the subject’s microbiome. We defined the following 11 clinical variables: short cervix: (YES/NO), cerclage (YES/NO), vaginal pH, BMI, progesterone (YES/NO), gravidity, parity, gravidity minus parity, history of miscarriage or stillbirth (YES/NO), history of PTB (YES/NO), use of antibiotics in 6 months before sampling. YES/NO features were converted to 1 = YES, 0 = NO. Other features were normalized to be in the 0–1 range by subtracting the minimal value, and then dividing by the difference between minimum and maximum in the dataset. The training of the clinical information-based predictive model operates in two stages, as with the microbiome-based model. First, features are filtered through a univariate Mann–Whitney *U*-test. Second, a regularized linear model is trained using features that remain after filtering. The leave-one-out estimates of the predictive power of the clinical information-based model are: AUROC 0.764, sensitivity 69.5%, specificity 74.2%.

### Cross-study comparison cohort selection

We performed a systematic literature review to identify previous studies that used taxonomic markers to assess the vaginal microbiome and PTB^22–27,30–37,57^. Three studies included cohorts of pregnant women who were predominantly of African descent, were at high risk for PTB and had publicly available 16S rRNA reads^25,27,30^ (bioProjects: PRJNA242473, PRJNA294119, PRJNA393472-(University of Alabama (UAB) cohort). De-identified clinical data for bioProject PRJNA294119 was kindly made available by Molly Stout. There was substantial variation in the inclusion and exclusion criteria for the case and control groups across studies. For example, Stout et al.^27^ excluded subjects taking supplemental progesterone from the study, whereas, in contrast, Callahan et al.^25^ included only subjects receiving supplemental progesterone in the UAB high-risk cohort. The inclusion criteria for PTB differed substantially; the study by Romero et al.^30^ included only participants who experienced spontaneous labor or preterm premature rupture of membranes and delivered before 34 weeks. In contrast, participants with indicated PTB (for example, pre-eclampsia) accounted for most PTBs in the Stout et al.^27^ study. There were similar differences in the control groups across studies. There were critical differences across all aspects of experimental design: sample collection, hypervariable region(s) of 16S rRNA examined, primer selection and PCR, sequencing technology and bioinformatics pipelines.

### Cross-study comparison cohort matching

In the present study, the selected cases were all spontaneous PTBs that occurred before 37 weeks of gestation; early term births (that is, those delivered between 37 weeks and 38 weeks 6 days) were excluded from the controls. To identify the most suitable replication cohorts available for the present study, we reanalyzed data generated from a subset of samples collected from women who experienced spontaneous PTB before 37 weeks of gestation and matched term controls delivered at a gestational age of 39 weeks or later. Only cases/controls with at least one sample, with a minimum of 1,000 reads after trimming and quality filtering, were included. Controls were matched 2:1 to cases from the same original study, based on the matching criteria used in the present study, as far as possible. This included ancestry/ethnicity for all three studies and age for two of the studies. Age was not available the Stout et al. study^27^ (that is, PRJNA294119). Annual household income was not used for matching because it was not available for any of the three studies. In the high-risk cohort from the Callahan et al.^25^ study (that is, PRJNA393472-UAB cohort), there was not a sufficient number of controls who met the criteria, and therefore cases for the available controls to preserve the matching ratio. For the Romero et al.^16^ study, there was an excess of available controls, so we restricted selection of controls to those who had a sample collected before 37 weeks of gestation. Selection of rematched cases/controls was performed blinded to other data.

### Replication cohort processing of 16S rRNA data

We applied a standardized bioinformatics pipeline to the 16S rRNA reads from the three original studies to harmonize with the present study as far as possible. Reads were downloaded from the SRA (bioProjects: PRJNA242473, PRJNA294119, PRJNA393472-UAB cohort). Cutadapt was used to trim adapters and primers, if present. We applied harmonized quality filtering across the studies. For studies using the 454 Roche sequencing technology, we applied filtering criteria established by our group in the Vaginal Human Microbiome Project which has been successfully used with our established STIRRUPS taxonomic classification^47^ and statistical analyses. Thus, we trimmed from the 3′-end of the read until the average quality score of the last 10 bp was 20 or more. If the resulting read was less than 200 bp in length, it was discarded. For the Stout et al.^27^ study, we analyzed only the V1–V3 reads. For the Callahan et al.^25^ study, the reads were trimmed and quality filtered, and forward and reverse reads were merged before upload to the SRA. The quality filtering criteria used for the Illumina HiSeq data in that original study (that is, each read of 235 bp or 245 bp needed to have fewer than two expected errors) was consistent with the filtering criteria applied to the Illumina MiSeq data in the present study (that is, MEEP score cutoff of 1). The forward and reverse reads both covered the V4 region, so we used only the forward reads from that study for taxonomic analysis. For all studies, only samples with a minimum of 1,000 quality-filtered reads were used in downstream analyses. A total of 85 of the 149 samples in the Stout et al. study met this QC threshold and 348 of 349 samples in the Romero et al.^16^ study. All 1,280 samples from the Callahan et al. UAB cohort^25^ had a minimum of 1,000 quality-filtered reads. Clinical data were available for two additional samples in the Callahan et al. UAB cohort for which sequence data were not available at the SRA.

### Cross-study comparison cohort taxonomic classification

Although the primers used in the Stout et al.^27^ and Romero et al.^16^ studies were different from each other and those used in the present study, the same V1–V3 hypervariable region was amplified. Thus, the trimmed quality-filtered reads from the Romero et al.^30^ and the Stout et al.^27^ studies were run through the STIRRUPS pipeline, using the same database as the present study. We do not have a curated custom database of vaginally relevant taxa for the V4 hypervariable region. Thus, we curated a small V4 database (https://github.com/Vaginal-Microbiome-Consortium/PTB) corresponding to our V1–V3 database for the taxa identified as very different between cases and controls in the present study. We used the STIRRUPS pipeline to align reads from the Callahan et al. UAB cohort^25^ against this small V4 database with taxa of interest.

Notably, in the Stout et al. replication cohort, all four taxa used in our predictive model (*S*. *amnii*, BVAB1, TM7-H1, *Prevotella* cluster 2) had higher abundance in the PTB group than in the TB group. Moreover, for 8 of the 14 taxa identified as differing between the PTB and TB groups in the analyses of our cohort, the difference in medians between the PTB and TB groups in the Stout cohort occurred with the same directionality as in our cohort. Of the remaining six taxa, four were infrequent and had medians below the abundance cutoff threshold in both TB and PTB groups of the Stout et al. cohort (that is, TM7-H1, BVAB2, *Dialister micraerophilus*, *P. amnii*), but they still had a higher abundance range in PTB than in TB groups, as in our cohort. Only Achr and Dcl51 showed an opposite trend of medians in the Stout et al. cohort compared with ours. In the Callahan et al. replication cohort, three of the four taxa used in our predictive model had a higher median in the PTB group than in the TB group, similar to our cohort. Moreover, in 11 of 14 taxa, the median between the PTB and TB groups in the Callahan et al. cohort differed in the same direction as in our cohort. TM7 had a median below the cutoff threshold. Only *Dialister* cluster 51 and *Megasphaera* type 1 OTU70 showed an opposite trend in the medians of the Callahan et al. cohort compared with ours.

### Nucleotide sequence accession numbers

The genome sequences of BVAB1 S1 (PQVO000000) and TM7-H1 E1 (CP026537) are available at the GenBank database.

### Reporting Summary

Further information on research design is available in the Nature Research Reporting Summary linked to this article.

## Online content

Any methods, additional references, Nature Research reporting summaries, source data, statements of code and data availability and associated accession codes are available at 10.1038/s41591-019-0450-2.

## Supplementary information

Supplementary Information Supplementary Tables 1–14 and Supplementary Fig. 1

Reporting Summary

Supplementary Data Supplementary Data 1–4

## Data Availability

Open-access data including raw 16S rRNA sequences, cytokine data and limited metadata are available at the HMP DACC (https://portal.hmpdacc.org). Controlled-access data including raw MGS data, raw MTS data and metadata for all subjects analyzed in this study are available at National Center for Biotechnology Information’s controlled-access dbGaP (study no. 20280; accession ID phs001523.v1.p1) and the SRA under BioProject IDs PRJNA326441, PRJNA326442 and PRJNA326441. The genomes of TM7-H1 (CP026537) and BVAB1 (PQVO000000) have been submitted to GenBank. Access to additional fields can be requested through the RAMS Registry (https://ramsregistry.vcu.edu). Additional project information is available at the project’s website (http://vmc.vcu.edu/momspi).

## Code availability

The custom code is available at https://github.com/Vaginal-Microbiome-Consortium/PTB.
