# Supplementary material for: The vaginal microbiome and preterm birth
Source: Nat Med. 2019 May 29;25(6):1012–21. doi: 10.1038/s41591-019-0450-2 (PMC6750801; doi:10.1038/s41591-019-0450-2)
Supplement: Supplementary file 1 — Supplementary Tables 1–14 and Supplementary Fig. 1 [file 41591_2019_450_MOESM1_ESM.pdf]

nature medicine
SUPPLEMENTARY INFORMATION
https://doi.org/10.1038/s41591-019-0450-2

In the format provided by the authors and unedited.

The vaginal microbiome and preterm birth

Jennifer M. Fettweis[1,2,3], Myrna G. Serrano[1,3], J. Paul Brooks[3,4], David J. Edwards[3,5], Philippe H. Girerd[2,3], Hardik I. Parikh[1], Bernice Huang[1], Tom J. Arodz[3,6], Laahirie Edupuganti[1,3], Abigail L. Glascock[7], Jie Xu[3,8,9], Nicole R. Jimenez[1,3], Stephany C. Vivadelli[1,3], Stephen S. Fong[3,10], Nihar U. Sheth[11], Sophonie Jean[1], Vladimir Lee[1,3], Yahya A. Bokhari[6], Ana M. Lara[1], Shreni D. Mistry[1], Robert A. Duckworth III[1], Steven P. Bradley[1], Vishal N. Koparde[11], X. Valentine Orenda[1], Sarah H. Milton[2], Sarah K. Rozycki[12], Andrey V. Matveyev[1], Michelle L. Wright[13,14,15], Snehalata V. Huzurbazar[16], Eugenie M. Jackson[16], Ekaterina Smirnova[17,18], Jonas Korlach[19], Yu-Chih Tsai[19], Molly R. Dickinson[1], Jamie L. Brooks[1], Jennifer I. Drake[1], Donald O. Chaffin[20], Amber L. Sexton[20], Michael G. Gravett[20,21], Craig E. Rubens[20], N. Romesh Wijesooriya[9], Karen D. Hendricks-Muñoz[3,8,9], Kimberly K. Jefferson[1,3], Jerome F. Strauss III[2,3] and Gregory A. Buck[1,3,6]*

[1]Department of Microbiology and Immunology, School of Medicine, Virginia Commonwealth University, Richmond, VA, USA. [2]Department of Obstetrics and Gynecology, School of Medicine, Virginia Commonwealth University, Richmond, VA, USA. [3]Center for Microbiome Engineering and Data Analysis, Virginia Commonwealth University, Richmond, VA, USA. [4]Supply Chain Management and Analytics, School of Business, Virginia Commonwealth University, Richmond, VA, USA. [5]Department of Statistical Sciences and Operations Research, College of Humanities and Sciences, Virginia Commonwealth University, Richmond, VA, USA. [6]Department of Computer Science, College of Engineering, Virginia Commonwealth University, Richmond, VA, USA. [7]VCU Life Sciences, Virginia Commonwealth University, Richmond, VA, USA. [8]Division of Neonatal Medicine, School of Medicine, Virginia Commonwealth University, Richmond, VA, USA. [9]Department of Pediatrics, School of Medicine, Children's Hospital of Richmond at Virginia Commonwealth University, Richmond, VA, USA. [10]Department of Chemical and Life Science Engineering, College of Engineering, Virginia Commonwealth University, Richmond, VA, USA. [11]Center for the Study of Biological Complexity, VCU Life Sciences, Virginia Commonwealth University, Richmond, VA, USA. [12]School of Medicine, Virginia Commonwealth University, Richmond, VA, USA. [13]Nell Hodgson Woodruff School of Nursing, Emory University, Atlanta, GA, USA. [14]Department of Women's Health, Dell School of Medicine, University of Texas at Austin, Austin, TX, USA. [15]School of Nursing, University of Texas at Austin, Austin, TX, USA. [16]Department of Biostatistics, School of Public Health, West Virginia University, Morgantown, WV, USA. [17]Department of Mathematical Sciences, University of Montana, Missoula, MT, USA. [18]Department of Biostatistics, School of Medicine, Virginia Commonwealth University, Richmond, VA, USA. [19]Pacific Biosciences, Menlo Park, CA, USA. [20]Global Alliance to Prevent Prematurity and Stillbirth, Seattle, WA, USA. [21]Department of Obstetrics & Gynecology, University of Washington, Seattle, WA, USA. *e-mail: gregory.buck@vcuhealth.org

Supplementary Table 1: Characteristics of the study cohort

	Preterm delivery < 37 wks (N=45)	Term delivery ≥ 39 wks (N=90)	
Mean BMI (kg/m2) †	28.9 (7.8)	30.9 (8.2)	
Pregnancy History:			
First conception	8/45 (17.8%)	24/90 (26.7%)	
Nulliparity	20/45 (44.4%)	30/90 (33.3%)	
Previous preterm births	14/45 (31.1%)	9/90 (10.0%)	**
Previous abortions‡	25/45 (55.5%)	32/90 (35.6%)	*
Infertility treatment	1/45 (2.2%)	3/89 (3.4%)	
Chronic hypertension	2/44 (4.4%)	4/90 (4.4%)	
Gestational hypertension	3/45 (6.7%)	6/90 (6.7%)	
Gestational Diabetes	1/45 (2.2%)	1/90 (1.1%)	
Chorioamnionitis	4/45 (8.9%)	8/90 (8.9%)	
Preeclampsia (not indication for PTB)	5/45 (11.1%)	4/90 (4.4%)	
Group B strep	20/37 (54%)	24/83 (28.9%)	*
Antibiotic usage§	23/45 (51.1%)	42/90 (46.7%)	
Smoking (current)	13/41 (31.7%)	15/87 (17.2%)	
Douching within past month	4/40 (10.0%)	1/69 (1.4%)	
ROM	44/44 (100%)	79/88 (89.8%)	
Short cervix	6/45 (13.3%)	0/90 (0%)	**
Cerclage	8/45 (17.8%)	0/90 (0%)	**
Progesterone	7/40 (17.5%)	4/86 (4.7%)	*
First vaginal sample self-collected	31/45 (68.9%)	60/90 (66.7%)	
More than 5 lifetime sexual partners	14/43 (32.6%)	41/87 (47.1%)	
Mean apgar score			
1 min	7.9 (2.1)	8.1 (1.9)	
5 min	8.1 (2.1)	8.7 (1.1)	

* Significant at p≤0.05(two-sided) by two-proportion Z-test

** Significant at p≤0.01(two-sided) by two-proportion Z-test

† Mean with standard deviation; BMI at first visit during pregnancy at which data was collected

‡ Abortions and miscarriages prior to 20 weeks gestation

§ Six months prior to enrollment through birth

Supplementary Table 2: Vagitype distribution and transitions

a) Summary of vagitypes (*i.e.,* community state types) observed in our preterm/term cohort

Vagitype	Preterm (N=45)	Term (N=90)
Lactobacillus iners	19 (42.22%)	34 (37.78%)
Lactobacillus crispatus	2 (4.44%)	18 (20%)
Lachnospiraceae BVAB1	7 (15.56%)	9 (10%)
Gardnerella vaginalis	3 (6.67%)	8 (8.89%)
Atopobium vaginae	2 (4.44%)	5 (5.56%)
Sneathia amnii	0 (0%)	2 (2.22%)
Prevotella cluster2	2 (4.44%)	0 (0%)
Streptococcus cluster29	0 (0%)	2 (2.22%)
"*Ca.* Mycoplasma girerdii"	1 (2.22%)	0 (0%)
Streptococcus agalactiae	1 (2.22%)	0 (0%)
Lactobacillus gasseri	1 (2.22%)	3 (3.33%)
Lactobacillus delbrueckii	0 (0%)	2 (2.22%)
No Type	7 (15.56%)	7 (7.78%)

* Vagitypes of first collected sample from 45 women who delivered preterm and 90 who delivered full-term.

b) Vagitype (*i.e.,* community state type) transition probabilities

**Preterm delivery**					
	G. vaginalis	BVAB1	*L. crispatus*	*L. iners*	Other
G. vaginalis	0.03	0.23	0.04	0.43	0.28
BVAB1	0.02	0.30	0.03	0.50	0.15
L. crispatus	0.00	0.01	0.88	0.03	0.08
L. iners	0.02	0.10	0.06	0.60	0.22
Other	0.03	0.14	0.06	0.35	0.42

**Term delivery**					
	G. vaginalis	BVAB1	*L. crispatus*	*L. iners*	Other
G. vaginalis	0.11	0.15	0.06	0.44	0.24
BVAB1	0.05	0.34	0.03	0.46	0.12
L. crispatus	0.02	0.01	0.82	0.06	0.09
L. iners	0.05	0.05	0.07	0.66	0.17
Other	0.08	0.08	0.10	0.47	0.27

***** Ninety-day transition probabilities for vagitypes derived from a Markov chain analysis with pregnancy outcome (preterm and term) included as a covariate.

Supplementary Table 3: Taxa abbreviations

Abbreviation	Taxon
Achr	*Aerococcus christensenii*
Avag	*Atopobium vaginae*
BVAB2	Clostridiales BVAB2
CL22	Clostridiales OTU22
CO27	Coriobacteriaceae OTU27
C45	*Corynebacterium* cluster 45
Dcl51	*Dialister* cluster 51
Dmic	*Dialister micraerophilus*
ENcl31	Enterobacteriaceae cluster 31
F48	*Fusobacterium* cluster 48
Gvag	*Gardnerella vaginalis*
Gmorcl	*Gemella morbillorum* cluster
G86	*Gemella* OTU86
Hcl60	*Haemophilus* cluster 60
BVAB1	Lachnospiraceae BVAB1
Lcol	*Lactobacillus coleohominis*
Lcricl	*Lactobacillus cripatus* cluster
Lgas	*Lactobacillus gasseri* cluster
Line	*Lactobacillus iners*
Ljen	*Lactobacillus jensenii*
Lvag	*Lactobacillus vaginalis*
Mty1/ Ms070	*Megasphaera* type1 OTU70
Mty2	*Megasphaera* type2 OTU71
Mhom	*Mycoplasma hominis*
P142	*Parvimonas* OTU142
Pben	*Porphyromonas bennonis*
Pamn	*Prevotella amnii*
Pbiv	*Prevotella bivia*
Pcl2	*Prevotella* cluster 2 (Note: contains *P. timonensis*)
P50	*Prevotella* cluster 50
Pcor	*Prevotella corporis*
P49	*Prevotella* OTU49
Ptim	*Prevotella timonensis*
PR61	Prevotellaceae OTU61
Samn	*Sneathia amnii*
Ssan	*Sneathia sanguinegens*
S47	*Staphylococcus* cluster 47
Sang	*Streptococcus anginosus*
Strepcl29	*Streptococcus* cluster 29
Ssalcl	*Streptococcus salivarius* cluster
TM7-H1	TM7-H1
Ucl23	*Ureaplasma* cluster 23

Supplementary Table 4: Comparison of abundance levels of bacteria in the first sample of the preterm and full term cohorts

Taxon	FDR-adjusted p-value PTB vs. TB<br>Mann-Whitney U test	Median abundance PTB cohort	Median abundance TB cohort	Abundance 75th-pctl PTB cohort	Abundance 75th-pctl TB cohort
Lactobacillus iners		0.16148	0.23891	0.72016	0.75417
Lactobacillus crispatus cluster	0.0325	0.00011	0.00028	0.00054	0.00905
Gardnerella vaginalis		0.08219	0.00401	0.15309	0.08559
Lachnospiraceae BVAB1	0.0278	0.00061	0.00006	0.09589	0.00064
Atopobium vaginae		0.01277	0.0002	0.05359	0.0163
Prevotella cluster2	0.0149	0.01214	0.00054	0.05699	0.00937
Lactobacillus gasseri cluster		0	0.00001	0.00006	0.0001
Lactobacillus jensenii		0.00003	0.00003	0.00147	0.00083
Megasphaera type1 OTU70		0.01032	0.00003	0.03086	0.01907
Sneathia amnii	0.0023	0.00071	0.00001	0.02149	0.00031
Dialister cluster51	0.0049	0.00307	0.00004	0.00813	0.00141
Prevotella amnii	0.0006	0.00018	0	0.00266	0.00002
Prevotella bivia		0.00004	0.00016	0.00031	0.00088
Ureaplasma cluster23		0.0002	0.00025	0.00212	0.00326
Aerococcus christensenii	0.0394	0.00083	0.00002	0.00487	0.00247
Coriobacteriaceae OTU27	0.0242	0.00198	0	0.00818	0.00241
TM7-H1		0	0	0.00006	0
Sneathia sanguinegens	0.0188	0.00026	0	0.00304	0.00011
Mycoplasma hominis		0.00003	0	0.00228	0.00005
Clostridiales BVAB2	0.007	0.00021	0	0.00424	0.0001
Dialister micraerophilus	0.0149	0.00225	0.0002	0.00414	0.0018
Megasphaera type2 OTU71		0	0	0	0
Prevotella cluster50		0.00001	0.00001	0.00214	0.00011
Prevotellaceae OTU61		0	0	0.00006	0
Finegoldia magna		0.00007	0.0001	0.00044	0.00055
Parvimonas OTU142	0.0081	0.00062	0	0.00351	0.00022

*Taxa with at least 5% of all subjects (n=135) having vaginal 16S rRNA abundance of 0.01 or higher, or at least 15% of subjects having abundance of 0.001 or higher. For each of these 26 abundant taxa, we provide median and 75-percentile of abundance levels in the PTB and TB cohorts, on the scale from 0 (not present) to 1 (100% of reads in the sample are from this taxa). We also provide the p-value (two-sided) for the Mann-Whitney U test for difference in abundance between the PTB and TB cohort, corrected using the Benjamini-Hochberg procedure with a False Discovery Rate of 5%; p-values are provided only for the taxa with statistically significant difference. The earliest sample available for each subject was used. Abundance values below 0.00001 were rounded down to 0.

Supplementary Table 5: Comparison of abundance levels of bacteria in the samples prior to 24 weeks of gestation in the preterm and full term cohorts

Taxon	FDR-adjusted p-value PTB vs. TB<br>Mann-Whitney U test	Median abundance PTB cohort	Median abundance TB cohort	Abundance 75th-pctl PTB cohort	Abundance 75th-pctl TB cohort
Lactobacillus iners		0.161479875	0.153030306	0.709926724	0.574055731
Lactobacillus crispatus cluster		0	0	0	0.517945364
Gardnerella vaginalis		0.118326016	0.003631449	0.182883576	0.148198731
Lachnospiraceae BVAB1	0.000751915	0.016490117	0	0.203617163	0
Atopobium vaginae		0.022259321	0	0.064351335	0.052899227
Prevotella cluster2	0.015110353	0.013067584	0	0.057595188	0.013028861
Lactobacillus gasseri cluster		0	0	0	0
Lactobacillus jensenii		0	0	0	0
Megasphaera type1 OTU70	0.039438636	0.016587732	0	0.033147332	0.015058744
Sneathia amnii	0.006076968	0.003664432	0	0.023375567	0
Dialister cluster51	0.010102959	0.004019905	0	0.009680544	0.001319003
Prevotella amnii	0.013544538	0	0	0.002351971	0
Prevotella bivia		0	0	0	0
Ureaplasma cluster23		0	0	0.002114076	0.003536482
Aerococcus christensenii		0	0	0.006053433	0.002639649
Coriobacteriaceae OTU27		0.002581544	0	0.011263814	0.002373542
TM7-H1	0.010102959	0	0	0.005823944	0
Sneathia sanguinegens		0	0	0.003490496	0
Mycoplasma hominis		0	0	0.002387851	0
Clostridiales BVAB2	0.047623929	0	0	0.005646437	0
Dialister micraerophilus	0.039438636	0.001793266	0	0.004399358	0.001751052
Megasphaera type2 OTU71		0	0	0	0
Prevotellaceae OTU61		0	0	0.00082111	0
Finegoldia magna		0	0	0	0
Parvimonas OTU142	0.039438636	0	0	0.005366583	0

*Taxa with at least 5% of subjects with samples collected within the first 24 weeks of pregnancy (n=90) having vaginal 16S rRNA abundance of 0.01 or higher, or at least 15% of subjects having abundance of 0.001 or higher. The earliest sample available for each subject was used. Abundance values below 0.001 were rounded down to 0. For each of these 25 abundant taxa, we provide median and 75-percentile of abundance levels in the PTB and TB cohorts, on the scale from 0 (not present) to 1 (100% of reads in the sample are from this taxa). We also provide the p-value (two-sided) for the Mann-Whitney U test for difference in abundance between the PTB and TB cohort, corrected using the Benjamini-Hochberg procedure with a False Discovery Rate of 5%; p-values are provided only for the taxa with statistically significant difference.

Supplementary Table 6: Genome characteristics of BVAB1 and TM7-H1

Organism	BVAB1 str, S1	TM7-H1 str. E1
Genome Size (Mb)	1.45	0.72
Phylum	Firmicutes	*Candidatus* Saccharibacteria
Family	Lachnospiraceae	Candidate division TM7*
Genus	ND	ND
N50 (Bp)	41,915	424,142
Contigs	22	3
DNA GC Content (%)	31.2	47.6
DNA coding region (%)‡	86.8	91.2
Total genes‡	1300	732
Average gene size (bp)‡	966	900
Coding sequences‡	1259	687
rRNA genes‖	n/a	3
tRNA genes‡	40	45
tmRNA genes‡	1	0
Episomal elements†	0	6
Repeat regions‡	1	0
Signal peptides‡	40	35
Hypothetical proteins‡	387	362
Virulence and defense determinants (hypothetical) †	120	17

* Unranked; ND, not defined.
† Identified using RAST (see Methods)
‡ Identified using Prokka (see Methods)
§ Identified using RNAmmer (see Methods)

Supplementary Table 7: Amino acid metabolism in metabolic models of L. crispatus, BVAB1 and TM7-H1

AMINO ACID PATHWAY	KEGG Reference	*L. crispatus*		BVAB1		TM7-H1	
		Count	% Complete	Count	% Complete	Count	% Complete
Alanine, Aspartate and Glutamate Metabolism	50	10	20	9	18	0	0
Glycine, serine and threonine metabolism	68	8	12	4	6	1	1
Cysteine and methionine metabolism	76	10	13	4	5	1	1
Valine, leucine and isoleucine degradation	36	1	3	0	0	0	0
Valine, leucine and isoleucine biosynthesis	14	0	0	0	0	0	0
Lysine biosynthesis	28	9	32	2	7	2	7
Lysine degradation	53	0	0	1	2	0	0
Arginine biosynthesis	28	3	11	7	25	0	0
Arginine and proline metabolism	86	0	0	3	3	1	1
Histidine metabolism	38	0	0	0	0	0	0
Tyrosine metabolism	61	1	2	1	2	0	0
Phenylalanine metabolism	66	1	2	2	3	1	2
Tryptophan metabolism	58	0	0	1	2	1	2
Phenylalanine, tyrosine and tryptophan biosynthesis	39	1	3	7	18	0	0

Supplementary Table 8: Carbohydrate metabolism in metabolic models of *L. crispatus*, BVAB1 and TM7-H1

CARBOHYDRATE PATHWAY	KEGG Reference	*L. crispatus*		BVAB1		TM7-H1	
		Count	% Complete	Count	% Complete	Count	% Complete
Glycolysis / Gluconeogenesis	47	11	23	12	26	9	19
Citrate cycle (TCA cycle)	25	2	8	0	0	0	0
Pentose phosphate pathway	55	8	15	10	18	6	11
Pentose and glucuronate interconversions	67	3	4	2	3	2	3
Fructose and mannose metabolism	75	6	8	7	9	5	7
Galactose metabolism	48	9	19	6	13	3	6
Ascorbate and aldarate metabolism	46	2	4	1	2	0	0
Starch and sucrose metabolism	77	7	9	5	6	4	5
Amino sugar and nucleotide sugar metabolism	119	13	11	12	10	6	5
Pyruvate metabolism	67	8	12	7	10	4	6
Glyoxylate and dicarboxylate metabolism	73	4	5	3	4	0	0
Propanoate metabolism	55	2	4	5	9	2	4
Butanoate metabolism	55	1	2	3	5	0	0
C5-Branched dibasic acid metabolism	22	0	0	0	0	0	0
Inositol phosphate metabolism	48	1	2	1	2	1	2

Supplementary Table 9: Taxonomic contribution of genes that significantly differ between preterm and term cohorts in metatranscriptomics data

	↑ Preterm	↑ Term
Prevotella cluster2	3820	1
Anaerococcus tetradius	1317	0
Peptoniphilus harei	1171	0
Megasphaera OTU70 type1	988	1
Prevotella cluster50	980	0
Prevotella disiens	975	0
Streptococcus anginosus	954	0
Sneathia amnii	909	0
Lachnospiraceae BVAB1	711	0
Dialister cluster51	690	0
Sneathia sanguinegens	569	0
Prevotella amnii	485	1
Prevotella bivia	406	0
Peptoniphilus duerdenii	309	1
Coriobacteriaceae OTU27	299	0
Gardnerella vaginalis	211	94
Dialister micraerophilus	191	0
Anaerococcus OTU147	180	0
Finegoldia magna	143	3
Mycoplasma hominis	132	0
Atopobium vaginae	122	4
Gemella OTU86	120	0
Prevotella corporis	81	11
Corynebacterium cluster45	78	20
Clostridiales OTU22	73	0
Campylobacter ureolyticus	35	0
Corynebacterium coyleae	35	1
Aerococcus christensenii	32	1
Lactobacillus coleohominis	32	103
Streptococcus agalactiae	21	0
Corynebacterium cluster58	16	5
Gemella morbillorum sanguinis haemolysans	15	0
Lactobacillus iners	15	2
Enterobacteriaceae cluster31	10	3
Fenollaria timonensis	10	0
Fusobacterium cluster48	10	0
TM7 OTU-H1	10	0
Porphyromonas bennonis	9	0
Corynebacterium aurimucosum nigricans	8	16
Megasphaera OTU71 type2	8	24
Peptoniphilus indolicus	7	0
Streptococcus cluster29	5	0
Staphylococcus cluster47	3	59
Actinomycetales OTU158	2	0
Lactobacillus gasseri cluster	2	65
Lactobacillus jensenii	2	27
Bifidobacterium breve cluster	1	24
Lactobacillus crispatus cluster	1	2327
Lactobacillus delbrueckii	0	14
Lactobacillus vaginalis	0	887
Ureaplasma cluster23	0	86

*Number of genes in taxa that significantly differ at padj<0.05 with a two-sided Wald test as implemented in DESeq2 and a Benjamini and Hochberg FDR correction.

Supplementary Table 10: Taxonomic contribution of genes that significantly differ between preterm and term cohorts in metagenomics data

	↑ Preterm	↑ Term
Prevotella cluster2	292	0
Mycoplasma hominis	55	0
Prevotella cluster50	29	0
Prevotella amnii	16	0
Sneathia sanguinegens	16	0
Prevotella corporis	12	0
Anaerococcus tetradius	11	0
Anaerococcus OTU147	10	0
Lachnospiraceae BVAB1	9	0
Prevotella bivia	9	0
Prevotella disiens	8	0
Sneathia amnii	6	0
Finegoldia magna	4	0
Peptoniphilus duerdenii	3	0
Aerococcus christensenii	2	0
Gemella morbillorum sanguinis haemolysans	2	0
Clostridiales OTU22	1	0
Coriobacteriaceae OTU27	1	0
Gardnerella vaginalis	1	605
Gemella OTU86	1	0
Lactobacillus iners	1	1
Megasphaera OTU71 type2	1	0
Peptoniphilus indolicus	1	0
Streptococcus anginosus	1	0
Streptococcus cluster29	1	0
Atopobium vaginae	0	1
Lactobacillus crispatus cluster	0	560

*Number of genes in taxa that significantly differ at padj<0.05 with a two-sided Wald test as implemented in DESeq2 and a Benjamini and Hochberg FDR correction.

Supplementary Table 11: Taxonomic contribution of genes predicted to be involved in bacterial secretion that significantly differ between preterm and term cohorts in metatranscriptomics data

	Secreted ↑ Preterm	Secreted ↑ Term
Prevotella cluster2	24	0
Sneathia amnii	15	0
Lachnospiraceae BVAB1	10	0
Megasphaera OTU70 type1	10	0
Sneathia sanguinegens	10	0
Prevotella cluster50	7	0
Prevotella disiens	6	0
Dialister cluster51	5	0
Dialister micraerophilus	3	0
Peptoniphilus harei	1	0
Lactobacillus crispatus cluster	0	2

*Number of genes predicted to be involved in bacterial secretion in taxa that significantly differ at padj<0.05 with a two-sided Wald test as implemented in DESeq2 and a Benjamini and Hochberg FDR correction.

Supplementary Table 12: Taxonomic contribution of genes predicted to be involved in bacterial secretion that significantly differ between preterm and term cohorts in metagenomics data

	Secreted ↑ Preterm	Secreted ↑ Term
Prevotella cluster2	8	0
Sneathia sanguinegens	3	0
Prevotella amnii	2	0
Sneathia amnii	2	0

*Number of genes predicted to be involved in bacterial secretion in taxa that significantly differ at padj<0.05 with a two-sided Wald test as implemented in DESeq2 and a Benjamini and Hochberg FDR correction.

Supplementary Table 13: Technical differences in protocols across studies of preterm birth and the vaginal microbiome

	Fettweis *et al.**	Stout *et al.*	Romero *et al.*	Callahan *et al.*†
BioProject	PRJNA430482	PRJNA294119	PRJNA242473	PRJNA430482
Collection Swab	BD BBL™ CultureSwabTM EZ swabs (double tipped)	Dual-tipped rayon swab (Starplex Scientific, Ontario, Canada)	Dacron swab (medical packaging swab – PAK™ Carmarillo, CA, USA)	Catch-All Sample Collection Swabs (Epicentre Biotechnologies)
Hypervariable region(s) of 16S rRNA	V1-V3‡	V1-V3‡;<br>V3-V5§	V1-V3‡	V4
Primers	4x - FP1 - AGAGTTYGATYMTGGCTYAG<br><br>1x - FP2 - AGARTTTGATCYTGGTTCAG<br><br>R - ATTACCGCGGCTGCTGG	27F - AGAGTTTGATCCTGGCTCAG<br><br>534R - ATTACCGCGGCTGCTGG	27F - AGAGTTTGATCCTGGCTCAG<br><br>4x - 27f-YM - AGAGTTTGATYMTGGCTCAG<br><br>1x - 27f-Bif - AGGGTTCGATTCTGGCTCAG<br><br>1x - 27f-Bor - AGAGTTTGATCCTGGCTTAG<br><br>1x - 27f-Chl - AGAATTTGATCTTGGTTCAG<br><br>534R - TTACCGCGGCTGCTGGCA	515F - AATTGTGTGYCAGCMGCCGCGGTAA<br><br>806R - GGACTACNVGGGTWTCTAAT
Sequencing Technology	Illumina MiSeq	Roche 454	Roche 454	Illumina HiSeq

* Current study
‡ The V1-V3 primers differ across all three studies
§ V3-V5 not used for replication cohort
† Only UAB cohort used in replication cohort

Supplementary Table 14: Comparison of preterm birth cases in studies of the vaginal microbiome and preterm birth

	Fettweis *et al.**	Stout *et al.*	Romero *et al.*	Callahan *et al.* (UAB cohort)	Callahan *et al.* (Stanford cohort)
African ancestry	35/45 (77.8%)	16/24 (66.7%)	17/18 (94.4%)	36/41 (87.8%)	0/9 (0%)
Mean BMI (kg/m^2)	28.9	30	25.7	30.0	28.1
Nulliparity	20/45 (44.4%)	--	7/18 (38.9%)	0/41 (0%)	2/9 (22.2%)
Previous Preterm	14/45 (31.1%)	17/24 (29.1%)	--	41/41 (100%)	--
Spontaneous Preterm Birth	45/45 (100%)	9/24 (37.5%)†	18/18 (100%)	37/41 (90%)	4/9 (44.4%)
PPROM	26/45 (57.8%)	3/24 (12.5%)	--	7/41 (17.1%)	2/9 (22.2%)
PTL	19/45 (42.2%)	6/24 (25%)	--	30/41 (73.2%)	2/9 (22.2%)
Preeclampsia (Indication for preterm)	0/45 (0%)‡	11/24 (45.8%)	0/18 (0%)	2/41 (0.05%)	2/9 (22.2%)
AHC§	4/45 (8.9%)	--	11/18 (61%)	--	--
Short Cervix	6/45 (13.3%)	--	--	--	--
Cerclage	8/45 (17.8%)	0/24 (0%)	--	--	--
Progesterone	7/40 (17.5%)	0/24 (0%)	--	41/41 (100%)‖	--

* Current study
† Six identified metadata provided by M. Stout; 5 had data that passed QC
‡ 5/45 (11.1%) PreE, but not indication for PTB
§ Acute Histologic Chorioamnionitis
‖ Intramuscular 17α-hydroxyprogesterone caproate therapy

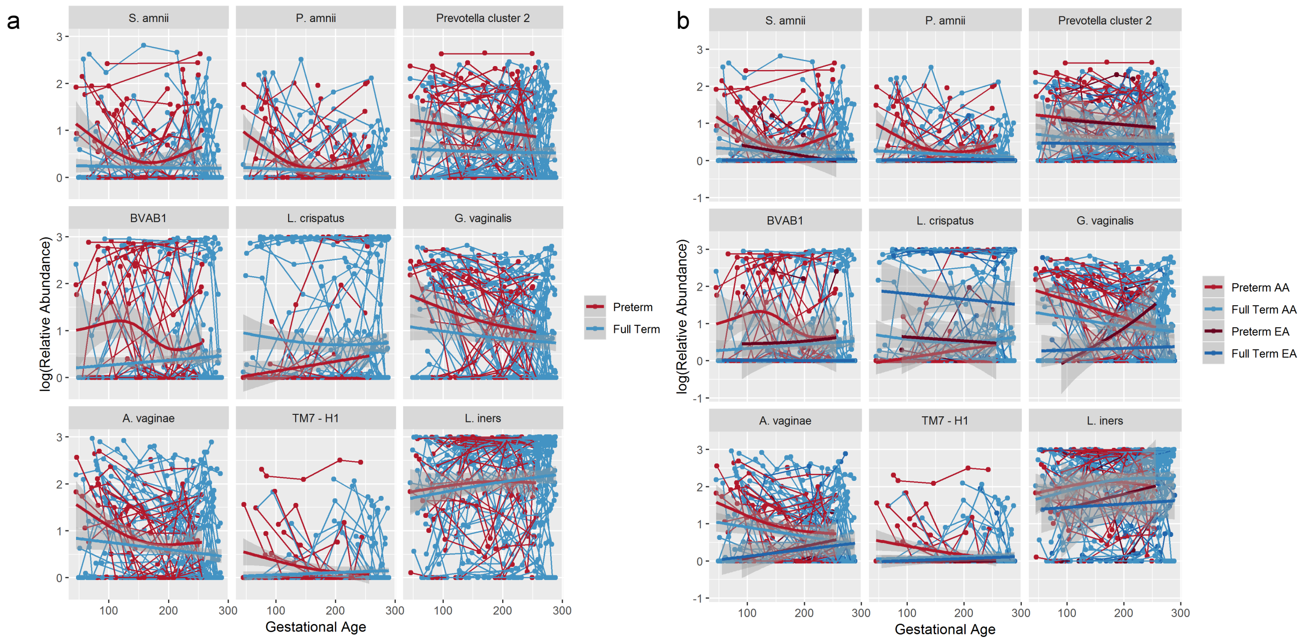

Supplementary Figure 1: Longitudinal generalized additive mixed effect model (GAMM) of vaginal microbiome composition during pregnancy. The model incorporates BMI, vaginal pH, pregnancy outcome (PTB, TB), a smoother for gestational age, and a random subject effect to longitudinally model log-transformed relative abundances of vaginally relevant taxa (see Methods). Each figure plots log-transformed abundances of taxa throughout pregnancy. Panel a exhibits plots comparing the preterm birth case (n=41) and full term birth cohorts (n=90). Panel b compares the results from preterm and full term women of African and European descent (*i.e.,* EA PTB n=7; AA PTB n=31;EA TB n=13;AA TB n=73). Confidence intervals (98%) are shown. Dots represent actual values.
