# Supplementary material for: The vaginal microbiome and preterm birth
Source: Nat Med. 2019 May 29;25(6):1012–21. doi: 10.1038/s41591-019-0450-2 (PMC6750801; doi:10.1038/s41591-019-0450-2)
Supplement: Supplementary file 2 — Reporting Summary [file 41591_2019_450_MOESM2_ESM.pdf]

nature research

Corresponding author(s): Gregory Buck

Last updated by author(s): Mar 18, 2019

Reporting Summary

Nature Research wishes to improve the reproducibility of the work that we publish. This form provides structure for consistency and transparency in reporting. For further information on Nature Research policies, see Authors & Referees and the Editorial Policy Checklist.

Statistics

For all statistical analyses, confirm that the following items are present in the figure legend, table legend, main text, or Methods section.

n/a	Confirmed	
☐	☒	The exact sample size (n) for each experimental group/condition, given as a discrete number and unit of measurement
☐	☒	A statement on whether measurements were taken from distinct samples or whether the same sample was measured repeatedly
☐	☒	The statistical test(s) used AND whether they are one- or two-sided<br>*Only common tests should be described solely by name; describe more complex techniques in the Methods section.*
☐	☒	A description of all covariates tested
☐	☒	A description of any assumptions or corrections, such as tests of normality and adjustment for multiple comparisons
☐	☒	A full description of the statistical parameters including central tendency (e.g. means) or other basic estimates (e.g. regression coefficient) AND variation (e.g. standard deviation) or associated estimates of uncertainty (e.g. confidence intervals)
☐	☒	For null hypothesis testing, the test statistic (e.g. F, t, r) with confidence intervals, effect sizes, degrees of freedom and P value noted<br>*Give P values as exact values whenever suitable.*
☐	☒	For Bayesian analysis, information on the choice of priors and Markov chain Monte Carlo settings
☐	☒	For hierarchical and complex designs, identification of the appropriate level for tests and full reporting of outcomes
☐	☒	Estimates of effect sizes (e.g. Cohen's d, Pearson's r), indicating how they were calculated

Our web collection on statistics for biologists contains articles on many of the points above.

Software and code

Policy information about availability of computer code

Data collection	Custom code will be available at https://github.com/Vaginal-Microbiome-Consortium/PTB prior to publication. Open source software is described in the test.
Data analysis	MeFiT for preprocessing of short read pair-end sequences, meep for quality filtering of paired-end sequences, QIIME for assignment of reads to Operational Taxonomic Units, Greengenes for 16S rRNA refrence sequences, UCHIME to screen for chimeric sequences, STIRRUPS using USEARCH for species-level classification of reads, bcl2fastq conversion software from Illumina for data demultiplexing, Adapter Removal tool v 2.1.3 to trim adapters, meeptools for quality trimming of reads, BWA for alignment of reads to reference sequences, MetaPhlAn2 for analysis of metagenomic and metatranscriptomic sequence, ASGARD, HUMAnN2 and ShortBRED for assignment of genes to pathways, BLAST for alignment of sequences, BBMap for normalizing of reads, SPAdes ver 3.8.0 for assembly of reads, Bowtie2 for alignment of reads to scaffolds, MyCC for clustering metagenomic contigs into specific taxonomic units, Newbler Assembler v 2.8 for assembly of reads, Prokka for gene annotation, HGAP for assembly of metagenomes from Pac Bio long read data, MacSysFinder was used to identify genes involved in bacterial secretion systems, FeatureCounts was used to count paired-end reads where both ends mapped to non-ribosomal genes, DESeq2 was used to compare term and preterm cohorts using an organism-independent global-scaling approach, MultiQuant software was used for analysis of lipidomic spectral data, various version of R were used for statistical profiling, REBACCA statistical tool to mitigate effects of relative constrain, Gephi for visualization of bacterial correlations, and a variety of custom scripts (deposited at GitHub) were used to generate the figures in the manuscript.

For manuscripts utilizing custom algorithms or software that are central to the research but not yet described in published literature, software must be made available to editors/reviewers. We strongly encourage code deposition in a community repository (e.g. GitHub). See the Nature Research guidelines for submitting code & software for further information.

Data

Policy information about availability of data

All manuscripts must include a data availability statement. This statement should provide the following information, where applicable:

- Accession codes, unique identifiers, or web links for publicly available datasets
- A list of figures that have associated raw data
- A description of any restrictions on data availability

Open-access data including raw 16S rRNA sequences, cytokine data and limited metadata are available at the HMP DACC (https://portal.hmpdacc.org/). Controlled-access data including raw metagenomic sequences, raw metatranscriptomic sequences and metadata for all subjects analyzed in this study are available at NCBI's controlled-access dbGaP (study number: 20280; accession ID: phs001523.v1.p1) and the Sequence Read Archive (SRA) under BioProject ID PRJNA326441. The genomes of TM7-H1(CP026537) and BVAB1 (PQVO000000) have been submitted to GenBank. Access to additional fields can be requested through the RAMS Registry (https://ramsregistry.vcu.edu). Additional project information is available at the project's website (http://vmc.vcu.edu/momspi).

Field-specific reporting

Please select the one below that is the best fit for your research. If you are not sure, read the appropriate sections before making your selection.

☒ Life sciences ☐ Behavioural & social sciences ☐ Ecological, evolutionary & environmental sciences

For a reference copy of the document with all sections, see nature.com/documents/nr-reporting-summary-flat.pdf

Life sciences study design

All studies must disclose on these points even when the disclosure is negative.

Sample size	In our initial power analysis (grant submission) we estimated with a Δ≥0.5, we would have power >98% with only 50 samples per group to identify differences between term and preterm samples. These were projected to include 'all' preterm births, including over 50% due to multiparous pregnancies and other medical reasons. In this project, we selected only 'spontaneous' preterm births, which have no obvious medical etiology. From the participants in the project, we were able to identify only 47 women who met all of our inclusion/exclusion criteria for spontaneous preterm births. These participants would have a higher delta than 'all' women who experience preterm birth. Moreover, several recent publications have used as low as 9 or fewer spontaneous preterm births, and no earlier publication had as many as 47. Thus, we believed this was a sufficient number to begin with. We were able to match these 47 with 94 term births to increase the statistical power of our analysis. In the end, we eliminated 2 preterm births (and their 4 controls) due to not having sufficient data.
Data exclusions	We started with 47 preterm births and 94 term births, but excluded 2 preterms and 4 terms because we lacked the 16S rRNA taxonomic data.
Replication	Experimental replication was not possible due to extensive cost. We attempted to replicate our findings using other data sets, with significant but not ideal results (see the manuscript). Sufficient data are not available in the community for such replication studies.
Randomization	All samples were randomized for sequencing and cytokine assay experiments. For 16S rRNA data, samples were randomized at the PCR stage and again at the sequencing stage.
Blinding	Case matching was performed blinded to all other study data.

Reporting for specific materials, systems and methods

We require information from authors about some types of materials, experimental systems and methods used in many studies. Here, indicate whether each material, system or method listed is relevant to your study. If you are not sure if a list item applies to your research, read the appropriate section before selecting a response.

Materials & experimental systems

n/a	Involved in the study	
☒	☐	Antibodies
☒	☐	Eukaryotic cell lines
☒	☐	Palaeontology
☒	☐	Animals and other organisms
☐	☒	Human research participants
☒	☐	Clinical data

Methods

n/a	Involved in the study	
☒	☐	ChIP-seq
☒	☐	Flow cytometry
☒	☐	MRI-based neuroimaging

Human research participants

Policy information about studies involving human research participants

Population characteristics	The population includes pregnant women 15 years and older and their neonates. Women selected for the the MOMS-PI Preterm study were predominantly of African ancestry (~78%) with a household income of less than $20,000 annually (76%).
Recruitment	Participants for this study were enrolled from women visiting maternity clinics in Virginia and Washington State. All study procedures involving human subjects were reviewed and approved by the institutional review board at Virginia Commonwealth University (IRB# HM15527). Participants were enrolled at multiple sites in Washington State by our partner registry, the Global Alliance to Prevent Prematurity and Stillbirth (GAPPS, www.gapps.org/). Study protocols were harmonized across sites, and data and samples from participants enrolled in Washington State were distributed to the VCU site. All study participants enrolled in Virginia and most participants enrolled at Washington State sites were also enrolled in the Research Alliance for Microbiome<br>3<br>nature research | reporting summary April 2018<br>Science (RAMS) Registry at Virginia Commonwealth University. RAMS Registry protocols were approved at Virginia Commonwealth University (IRB# HM15528); GAPPS-associated sites ceded review to the VCU IRB through reliance agreements. The study was performed with compliance to all relevant ethical regulations. Written informed consent was obtained from all participants and parental permission and assent was obtained for participating minors at least 15 years of age.<br>Pregnant women were provided literature on the project and invited to participate in the study. Women who: i) were incapable of understanding the informed consent or assent forms, or ii) were incarcerated were excluded from the study. Comprehensive demographic, health history and dietary assessment surveys were administered, and relevant clinical data (e.g., gestational age, height, weight, blood pressure, vaginal pH, diagnosis, etc.) was recorded. Relevant clinical information was also obtained from neonates at birth and discharge.<br>At subsequent prenatal visits, triage, in labor and delivery, and at discharge, additional surveys were administered, relevant clinical data was recorded and samples were collected. Vaginal and rectal samples were not collected at labor and delivery or at discharge. Women with any of the following conditions were excluded from sampling at a given visit:<br>1. Incapable of self-sampling due to mental, emotional or physical limitations.<br>2. More than minimal vaginal bleeding as judged by the clinician.<br>3. Ruptured membranes prior to 37 weeks.<br>4. Active herpes lesions in the vulvovaginal region.<br>Case/control design. We selected 47 preterm cases of singleton, non-medically indicated preterm births from women who delivered between 23 weeks 1 day and 36 weeks 6 days gestation and were enrolled in the Virginia arm of the study and delivered at the site prior to August 2016. From this cohort of 627 women, 82 delivered prior to 37 weeks. Tweleve of the participants who delivered preterm had multiple gestation pregancies, 21 experienced medically indicated delivery, one delivered following fetal demise and one delivered a fetus at a non-viable gestational age. The participants had completed the study through delivery, and their gestational age information had been recorded in the study operational database as of July 2016. We case-matched the preterm participants 2:1 with participants who completed the study with singleton term deliveries ≥39 weeks to avoid complications associated with early term birth51–53, matching based on ethnicity, age and income. With these criteria, we matched controls to cases as close as possible, loosening criteria at each pass using an in-house script; a few difficult-to-match cases were matched by hand. Case matching was performed blinded to all other study data. Two of the 47 preterm births did not have 16S rRNA that passed QC, thus these PTB samples and their controls were excluded from the taxonomic 16S rRNA analyses (Fig. 1) and demographic data in Table 1.<br>Possible self-selection bias. Women were approached in our prenatal clinics, informed of the project with its goals and protocols, and asked if they would like to participate. Since women were given the choice to participate, there is always the possibility that there could be self selection bias. That said, we detected no such bias, as most women were motivated to participate as they were quite aware of the challenges of adverse events in pregnancy and preterm birth.
Ethics oversight	Institutional Review Board for Human Subjects REsearch at VCU

Note that full information on the approval of the study protocol must also be provided in the manuscript.
